# Development of Novel Promiscuous Anti-Chemokine Peptibodies for Treating Autoimmunity and Inflammation

**DOI:** 10.3389/fimmu.2017.01432

**Published:** 2017-11-23

**Authors:** Michal Abraham, Hanna Wald, Dalit Vaizel-Ohayon, Valentin Grabovsky, Zohar Oren, Arnon Karni, Lola Weiss, Eithan Galun, Amnon Peled, Orly Eizenberg

**Affiliations:** ^1^Biokine Therapeutics Ltd, Ness Ziona, Israel; ^2^Department of Neurology, Tel Aviv Sourasky Medical Center, Tel Aviv, Israel; ^3^Sackler Faculty of Medicine, Tel Aviv University, Tel Aviv, Israel; ^4^Goldyne Savad Institute of Gene Therapy, Hebrew University of Jerusalem, Jerusalem, Israel

**Keywords:** chemokines, autoimmunity, inflammation, phage display, peptibodies

## Abstract

Chemokines and their receptors play critical roles in the progression of autoimmunity and inflammation. Typically, multiple chemokines are involved in the development of these pathologies. Indeed, targeting single chemokines or chemokine receptors has failed to achieve significant clinical benefits in treating autoimmunity and inflammation. Moreover, the binding of host atypical chemokine receptors to multiple chemokines as well as the binding of chemokine-binding proteins secreted by various pathogens can serve as a strategy for controlling inflammation. In this work, promiscuous chemokine-binding peptides that could bind and inhibit multiple inflammatory chemokines, such as CCL2, CCL5, and CXCL9/10/11, were selected from phage display libraries. These peptides were cloned into human mutated immunoglobulin Fc-protein fusions (peptibodies). The peptibodies BKT120Fc and BKT130Fc inhibited the ability of inflammatory chemokines to induce the adhesion and migration of immune cells. Furthermore, BKT120Fc and BKT130Fc also showed a significant inhibition of disease progression in a variety of animal models for autoimmunity and inflammation. Developing a novel class of antagonists that can control the courses of diseases by selectively blocking multiple chemokines could be a novel way of generating effective therapeutics.

## Introduction

Chemokines have been shown to be selective chemo-attractants for leukocyte sub-populations *in vitro* and to elicit a selective accumulation of immune cells *in vivo*. In addition to chemotaxis, chemokines mediate leukocyte degranulation (1) and adhesion receptor upregulation (2).

Chemokines are small (5–20 kDa, ~70–90 amino acids) proteins that are rich in basic amino acids and contain conserved cysteine motifs that form essential disulfide bonds between the first and third as well as the second and fourth cysteine residues. The numbers and spacing of the first cysteine residues in the amino acid sequence are used to classify chemokines into four subfamilies: CXC (or α), CC (or β), CX 3 C, and C chemokines. Currently, 20 chemokine receptors mediate the effects of more than 50 known chemokines (3). Interestingly, chemokines and their receptors have overlapping specificities for each other, unlike other types of G protein-coupled receptors. For this reason, the chemokine system is often thought to show significant redundancy, as one receptor can bind multiple ligands, and conversely, a single ligand can bind several chemokine receptors (4, 5).

Chemokines and their receptors have received increased attention due to their critical roles in the progression of inflammation and associated conditions, such as asthma, atherosclerosis, graft rejection, AIDS, autoimmune conditions (e.g., multiple sclerosis, arthritis, myasthenia gravis, lupus, and AMD) and cancer (3). The role of chemokines has been thoroughly investigated in pathogen conditions, such as rheumatoid arthritis (RA) and multiple sclerosis (MS). RA is a chronic inflammatory disease that eventually leads to joint destruction. RA is characterized by the infiltration of neutrophils, T and B lymphocytes, and macrophages into the synovial membrane and fluid compartment (6). The infiltrating cells, such as macrophages, T cells, B cells, and dendritic cells, play important role in the pathogenesis of RA. Chemokines have an important role in the pathogenesis of RA by recruiting leukocytes and by controlling other important processes, such as release of mediators of inflammation, cell proliferation, and angiogenesis. Synovial fluid from actively involved joints contains a number of chemokines, including CCL2, CCL3, CCL5, CXCL8, and CXCL10. Both synovial-lining cells and infiltrating cells are sources of these chemokines. The receptors CCR2, CCR5, CXCR2, and CXCR3 have been detected in infiltrating cells. MS is an inflammatory, demyelinating disease of the central nervous system (CNS), suspected to be of autoimmune origin, in which CNS myelin proteins serve as autoantigens. MS is characterized by the recruitment of T lymphocytes (predominantly Th1/Th17) and macrophages into the CNS. Infiltration of these cells into the CNS contributes to the disease pathology and is mediated by several chemokines. The chemokines that were found to be involved in the pathogenesis of MS were CCL5, CCL2, CCL3, CCL4, CXCL9, CXCL11, and CXCL10 (7, 8).

Despite the extensive involvement of chemokines in many pathological conditions, except for selective CCR5 antagonists for HIV and a selective CXCR4 antagonist for stem cell mobilization, obtaining new therapeutics related to targeting single chemokine receptors and chemokines against autoimmunity, therapies for inflammation and cancer has been unsuccessful (5, 9). The lack of significance clinical results in these studies could be due to a variety of reasons, including the lack of predictability of animal models for inflammatory and autoimmune diseases, relevance of the target to the human disease, genetic diversity between the tested patient population, unsuitable PK/PD properties of the drug candidate which lead to reduced chemokine receptor coverage, ineffective dosing, and the redundancy of the chemokine/chemokine receptor system (4, 5).

Binding to multiple chemokines as a strategy for inhibiting inflammation is a natural phenomenon. As quoted by Heidarieh et al. (10), atypical chemokine receptors, such as ACKR1, ACKR2, or ACKR4, that bind multiple chemokines, do not transduce signals function as chemokine scavengers to control the magnitude of inflammation (11). Furthermore, pathogens such as herpesviruses, ticks, and the trematode *Schistosoma mansoni* secrete chemokine-binding proteins (CKBPs) that also inhibit inflammatory chemokines and inflammation (12).

The aim of this study was to use phage display peptide libraries to identify and develop novel promiscuous anti-chemokine-binding peptides to inhibit autoimmune and inflammatory disease progression. Since peptides are usually rapidly cleared due to the combined effects of having poor metabolic stability and hydrodynamic radii that fall below the limit of kidney glomerular filtration, we choose to present these peptides on mutated human Fc domain which have reduced antibody-dependent cell-mediated cytotoxicity (ADCC) and mitogenicity properties (13–15). This approach retains certain desirable antibody features, including increased apparent affinity through the avidity conferred by the dimerization of two Fcs and a prolonged PK. Peptide–Fc fusion or peptibodies are an attractive alternative therapeutic format to monoclonal antibodies and have already been used in the clinic (16).

## Materials and Methods

### Materials

Amplification buffer was obtained from Invitrogen (CA, USA); Dextran T-500 was obtained from Pharmacosmos A/S (Denmark); Dulbecco’s Modified Eagle’s Medium (DMEM) was obtained from Biological Industries (Beit Haemek, Israel); ECL solution was obtained from Amersham Biosciences (Buckinghamshire, UK); EcoRI was obtained from New England Biolabs; enhancer solution was obtained from Invitrogen; EX-CELL^®^ 293 medium was obtained from SAFC Biosciences; fetal calf serum (FCS) was obtained from Biological Industries (Beit Haemek, Israel); Ficoll 1077 was obtained from Sigma-Aldrich (Israel).

Freund’s complete adjuvant (CFA) was obtained from Sigma-Aldrich or Difco; FuGENE^®^ 6 was obtained from Roche; methylated bovine serum albumin (mBSA) was obtained from Sigma-Aldrich; NotI was obtained from New England Biolabs; NuPAGE^®^ Bis-Tris gels were obtained from Invitrogen; the pIRESpuro3 vector was obtained from BD Biosciences Clontech; Protein A-Sepharose^®^ beads were obtained from Amersham; RPMI medium was obtained from Biological Industries (Beit Haemek, Israel); Taq polymerase (Platinum^®^ Pfx DNA polymerase) was obtained from Invitrogen; VCAM-1 (human) was obtained from R&D Systems, Inc. (Minneapolis, MN, USA); and all the recombinant human chemokines were ordered from PeproTech, Inc. (Rocky Hill, NJ, USA).

### Phage Selection

Phage display libraries (Ph.D-12™ and Ph.D-C7C Peptide LibrariesKits) were purchased from New England Biolabs (Beverly, MA, USA). Peptides were fused to the N-terminus of the M13 phage gene III protein with a GGGS spacer.

For phage selection, 60 × 15 polystyrene Petri dishes were coated with CCL11, CXCL8, CXCL12, CXCL9, and CCL2 (1 ml, 0.1–1 µg/ml in 0.1 M NaHCO_3_, pH 8.6) overnight at 4°C or 3–6 h at room temperature (RT) with gentle agitation in a humidified container. Coating the plates with the chemokine resulted in an immobilized, but still active (i.e., capable of binding), chemokine. The plates were blocked with BSA (5 mg/ml) in 0.1 M NaHCO_3_ for 1 h at RT or overnight at 4°C. The plates were washed six times with TBST (50 mM Tris–HCl (pH 7.5), 150 mM NaCl, 0.1% Tween), and incubated for 1 h at RT with the phage suspension [1 ml containing 10^9^–10^11^ plaque forming units (PFU)]. Unbound phages were removed by washing with TBST 10 times. After each round of selection, bound phages were eluted with 1 ml of 0.2 M glycine-HCl (pH 2.2) and 1 mg/ml BSA for 10 min at RT with gentle rocking. Eluted phages were immediately neutralized in 150 µl of 1 M Tris–HCl (pH 9.1). Eluted phages were titered to assess the number of phages that were bound to the plate and then amplified by infection with an overnight ER2738 *Escherichia Coli* culture in LB medium (diluted 1:100) for 4.5 h at 37°C with vigorous shaking. Phages were obtained by double precipitation with a 1:5 polyethylene glycol (PEG)-NaCl solution (20% PEG, 2.5 M NaCl) and dissolved in TBS (50 mM Tris–HCl (pH 7.5), 150 mM NaCl) to a final concentration of ~10^12^ PFU/ml. In total, 3 or 4 rounds of selection were performed. Single phages from the third or fourth selection were analyzed for their ability to specifically bind the chemokine of interest using ELISA. The specific peptides were DNA sequenced.

### Phage Titration

For phage titration, 200 µl of mid-log phase (OD_600_~0.5) EΠ*2738 Escherichia Coli* cultures were infected with 10 µl of 10-fold serial phage dilutions in TBS for 1–5 min at RT. The infected cells were transferred to culture tubes containing 4 ml of 45°C melting top-agar and immediately poured onto pre-warmed LB/IPTG/X-Gal-coated plates. The phages were incubated overnight at 37°C. PFU values were calculated by counting the blue plaques that appeared on the plates.

### ELISA for Phages

NUNC-immuno maxisorp plates were coated with the appropriate chemokine (0.1 ml/well, 0.1–1.0 µg/ml in 0.1 M NaHCO_3_, pH 8.6) for 3 h at RT or overnight at 4°C. The plates were then blocked with 5 mg/ml BSA (0.2 ml/well) in 0.1 M NaHCO_3_ (pH 8.6) overnight at 4°C or for 1 h at RT. Control wells were treated with blocking buffer alone with no target protein added. The plates were washed six times with TBST and then incubated with individually eluted phages (each representing individual peptides, 10^7^–10^9^ PFU in 0.1 ml of blocking buffer/well) for 45 min at RT. After the plates were washed six times with TBST, the bound phages were probed with an HRP/anti-M13 monoclonal conjugate [horseradish peroxidase conjugated to a mouse anti-M13 monoclonal antibody (mAb, Amersham Pharmacia Biotech, diluted 1: 5,000 to 1: 10,000 in blocking buffer, 0.1 ml/well)] for 45 min at RT. Target-bound phages were determined using a DAKO TMB one-step substrate system [3,3′,5,5′-tetramethlbenzidine, DAKO, CA USA (100 µl/well)]. The reaction was stopped by adding an HCl–H_2_SO_4_ mixture (1 N HCl, 3 N H_2_SO_4_). The results were analyzed by an ELISA reader (Anthos Labtec HT2, version 1.06) at OD_450_.

### DNA Purification and Sequence Analysis

In total, 10^12–13^ phages were amplified by infecting an overnight ER2738 *Escherichia Coli* culture diluted 1:100 in 10 ml of LB medium for 4.5 h at 37°C while vigorously shaking. Phages were obtained by precipitating with a 1:5 PEG–NaCl solution (20% PEG, 2.5 M NaCl). Single-stranded DNA (soda) was extracted by thoroughly suspending the phage pellet in 300 µl of iodide buffer (10 mM Tris–HCl (pH 8.0), 1 mM EDTA, 4 M NaCI) and then pelleting for 10 min. The pellet was incubated with 750 µl of ethanol and then centrifuged at RT for 10 min at 14,000 g. After being washed with cold 70% EtOH, the ssDNA pellet was re-suspended in 30 µl of water (double processed tissue culture water) (Sigma-Aldrich Co., Irvine KA, UK), and 0.5 µg of ssDNA was sequenced with a 10 pmol primer (5′-CCCTCATAGTTAGCGTAACG-3′, -96glll sequencing primer, New England Biolabs) by the Sequencing Unit at the Weizmann Institute of Science in Israel.

### Peptide Synthesis

Peptides were synthesized at the Weizmann Institute of Science in Rehovot, Israel to perform characterization tests on their ability to influence the biological activity of chemokines. The format of the various synthesized peptides was as follows: Cyclic peptides (ACX_7_CGGGSK-biotin-G) and linear peptides (X_12_GGGSK-biotin-G) were used. The peptides were biotinylated at their C-termini; biotin served as the detector during the subsequent experiments. Each synthetic peptide was dissolved to a final concentration of 1 mg/ml (~0.6 mM) in 4% DMSO (dimethyl sulfoxide, Sigma).

### ELISA of Synthetic Chemokine-Binding Peptides (CBPs)

NUNC-immuno maxisorp plates were coated with the appropriate chemokine (0.1 ml/well, 0.1–1.0 µg/ml in 0.1 M NaHCO_3_, pH 8.6) and incubated overnight at 4°C. The plates were then blocked with 0.2 ml/well of 5 mg/ml BSA in 0.1 M NaHCO_3_ (pH 8.6) for 3 h at RT. Control wells were treated with blocking buffer alone with no target protein added. The plates were washed six times with TBST, followed by incubation with 10-fold serial dilutions of the peptides (10 µg–10 pg in 0.1 ml TBST with 5 mg/ml BSA (TBST-BSA)/well) for 45 min at RT. After the plates were washed six times with TBST, the bound peptides were probed with either an HRP-SA conjugated antibody [horseradish peroxidase conjugated to streptavidin (R&D systems, diluted 1: 10,000 to 1: 20,000 in TBST–BSA, 0.1 ml/well) or AP-anti-biotin (alkaline phosphatase conjugated to a monoclonal anti-biotin antibody, clone BN-34, Sigma, diluted 1: 2,500 to 1: 5,000)] for 45 min at RT. The target-bound peptides that were probed with HRP-SA were determined using a DAKO TMB one-step substrate system (3,3′,5,5′-tetramethlbenzidine, DAKO) (100 µl/well). The reaction was stopped by the addition of an HCl–H_2_SO_4_ mixture (1 N HCl, 3 N H_2_SO_4_). The results were analyzed by an ELISA reader (Anthos Labtec HT2, version 1.06) at OD_450_. The target-bound peptides that were probed with AP-anti-biotin were determined using a Sigma 104 phosphate substrate [(p-nitrophenyl phosphate, disodium, hexahydrate), 5 mg tablets, Sigma]. The 5-mg Sigma 104 tablets were dissolved in 10 ml of developing buffer (50 mM Na_2_CO_3_, 0.2 mM MgCl_2_, pH 9.8) (100 µl/well). The results were analyzed by an ELISA reader (Anthos Labtec HT2, version 1.06) at OD_405_.

### Western Blot

Cell sample media were collected and centrifuged (1,000 rotations per minute, 7 min), and 1 ml of cell-deprived media was incubated with 50 µl of Protein A-Sepharose^®^ beads for 45 min at RT. The proteins were then eluted from the beads with 50 µl of sample buffer containing 100 mM citrate phosphate buffer (pH 3.5) and 10 mM DTT (dithiothreitol). The samples were boiled for 3 min, and 25 µl of the samples was then loaded onto 12% NuPAGE^®^ Bis-Tris gels. The proteins were transferred to nitrocellulose membranes and blocked with 10% low-fat milk in PBST (PBS supplemented with 0.05% Tween-20). The membranes were then blotted for 1 h with a human anti-IgG Fc fragment antibody followed by a goat anti-human antibody conjugated to HRP (horseradish peroxidase) diluted 1: 40,000 in blocking solution at RT. Following incubation with an ECL solution, the membrane was exposed to film.

### Cloning the Peptides into Fc–Peptides (Peptibodies)

#### Two-Step PCR for Obtaining IL6-BKT Peptide DNA Fragments

In the first PCR step, the IL6 signal peptide DNA sequence was added to the DNA sequence corresponding to the first nine amino acids of each peptide. The product of this PCR was used in the second PCR step as the template to which a DNA sequence corresponding to the remaining three amino acids of the peptide, a spacer sequence, and a 3′-end BstBI restriction site was added.

IL6-Fc pIRES puro DNA served as the DNA template for all three of the first-step PCR. All three PCR were performed using the same forward primer (T7). Using the universal T7 primer resulted in a PCR product that contained multiple cloning sites at the 5′ end, including an NheI site, which was used for later sub-cloning. The reverse primers were specific for each peptide; ctgtggatgc tgtggctggg gatgctgggg ggcacggggg caggg was used for BKT130, cacggtctga gggatagggt agtccagggg gggcacgggg gcagg was used for BKT120, and gtcgtagtcgaaagaaggtggtcacgggggcacgggggcagg. was used for BKT110Fc. BKT110 VTTFFDYDYGAPC-FC peptide was derived from the CCR2 n-terminal region and was used as a control peptibody.

The PCR conditions were as follows: 20 ng of DNA template, 5 µl of amplification X10 buffer, 0.2 mM of each deoxyribonucleotide (dNTP), 0.5 mM MgSO_4_, 5 µl of X10 enhancer solution, 0.2 µM of each primer, 2.5 units of Taq polymerase, and water added to a total reaction volume of 50 µl.

Amplifications were performed with an initial denaturation step at 94°C for 3 min, followed by 30 cycles of 94°C for 30 s, 52°C for 30 s, and 72°C for 30 s, and a final step at 72°C for 10 min.

At the end of PCR amplification, 5 µl of each reaction mixture was analyzed on 2% agarose gels stained with ethidium bromide and visualized with UV light.

The PCR products obtained by the above procedures were used as templates for the second PCR step. The abovementioned T7 primer was used as the forward primer in all three reactions, while the reverse primer was specific for each peptide; SEQ cgcttcgaag cccttgctgc cgccgccggc cttggcgctg tggatgctgt ggctgg was used for BKT130, SEQ cgcttcgaag cccttgctgc cgccgccgtg gtgcagcacg gtctgaggga taggg was used for BKT120 and SEQ cgcttcgaagcccttgccgccgccgcatggggcgccgtagtcgtagtcgaagaagg was used for BKT110. Each reverse primer encoded amino acids 10–12 of the desired peptide, a hexapeptide spacer sequence (Gly Gly Gly Ser Lys Gly) and a BstB1 restriction site.

The PCR conditions were as follows: 0.5 µl of the DNA template; 5 µl of X10 amplification buffer, 0.2 mM of each dNTP, 0.5 μM MgSO_4_, 5 µl of X10 enhancer solution, 0.2 µM of each primer, 2.5 units of Taq polymerase, and water added to a total reaction volume of 50 µl.

Amplifications and analysis of the PCR products were performed as described above for the first PCR step. After PCR amplification, the products were analyzed on 2% agarose gels stained with ethidium bromide and visualized with UV light. The PCR products were extracted from gels using a QiaQuick™ Gel Extraction kit (Qiagen™).

#### Ligation of IL6-BKT Peptide DNA Fragments to the Fc Sequence

Extracted IL6-BKT peptide DNA products were digested with NheI and BstBI, purified from agarose gels as described above, and ligated into Fc N297A pIRESpuro3 that was previously digested with the same enzymes.

This N297A Fc is a well-known mutation that prevents glycosylation and has a markedly decreased capacity to bind Fc receptors. This humanized Fc was found to be not mitogenic and circumvent the side effects that are linked to mitogenicity and ADCC (13–15).

The ligation mixture was then transformed into DH5a competent cells. Ampicillin-resistant transformants were screened, and positive clones were further analyzed by colony PCR.

DNA was extracted from ampicillin-resistant colonies and subjected to a 2-h digestion at 37°C with the EcoRI and NotI restriction enzymes. The reaction was resolved on an agarose gel. Positive colonies were expected to yield two bands corresponding to 5,123 and 868 bp. The positive colonies were verified by DNA sequencing.

#### Transfection

The constructs were transfected into HEK-293T cells using FuGENE 6 (Roche). Each BioPep construct was transfected into two wells of a 6-well plate. One well was dedicated for Western blot (WB) analysis, and the second well was dedicated for the establishment of a stable pool. The cells were plated in 6-well plates at a concentration of 500,000 cells per well. A day later, the cells were transfected with 6 µl of FuGENE and 2 µg of DNA (ratio of). Media from the wells that were dedicated for WB was replaced with serum-free media after 24 h and collected 48 h later. Media in the other wells was replaced with DMEM containing 10% FCS, and 48 h later, the cells were trypsynized and transferred to a T75 flask containing selection medium (DMEM, 10% FCS and 5 µg/ml puromycin) to obtain stable clones.

### T Cell Purification from Fresh Blood

Briefly, 50 ml of blood was added to 10 ml of dextran [Dextran T-500, 6% w/v in phosphate buffered saline (PBS)] and 7 ml of citrate buffer (25 g citrate and 8 g citric acid in 500 ml of PBS). The solution was incubated for 30 min at 25°C and separated using Ficoll 1077 (Sigma). The interphase was collected and washed twice with 8 ml of PBS-5% FCS, followed by centrifugation at 1,400 rpm for 5 min at 18°C. The cells were re-suspended in PBS-5% FCS at a concentration of less than 10^8^/ml. Next, 2 ml of the cell solution was applied, and the mixture was incubated for 45 min at 25°C on a Perspex nylon wool column, which was pre-soaked in PBS-5% FCS. Each column was washed with 8 ml of PBS-5% FCS, and the cells (T cells and erythrocytes) were eluted using 50 ml of 5 mM EDTA in PBS. A red pellet was obtained by centrifugation at 1,400 rpm for 5 min at 4°C. To lyse the erythrocytes, the red pellet was re-suspended in 5 ml of lysis buffer (155 mM NH_4_Cl, 10 mM KHCO_3_, 0.1 mM EDTA, 0.1 M PBS) for 4 min, followed by the immediate addition of 50 ml of PBS–EDTA.

Following centrifugation at 1,400 rpm at 4°C for 5 min, the pellet was washed again with 50 ml of PBS–EDTA and re-centrifuged under the same conditions. The pellet was re-suspended in RPMI/10% FCS/L-glutamine/sodium pyruvate/antibiotics at a concentration of 3 × 10^6^ cells/ml. The cells were incubated for 2 h at 37°C, and the non-adherent cells were collected. The cells were ready for use in experiments after overnight incubation at 37°C.

### Preparation of Adhesive Subsites

Human VCAM-1 (1 µg/ml) and CXCL12 (intact or heat-inactivated) (2°μg/ml) were dissolved in PBS buffered with 20 mM bicarbonate (pH 8.5) and incubated on polystyrene plates overnight at 4°C. The plates were then washed three times and blocked with human serum albumin (HAS) (20 µg/ml in PBS) for 2 h at 37°C.

### Biovalidation

#### Adhesion Assay Using Laminar Flow

Laminar flow assays were performed as described below. Polystyrene plates (Becton Dickinson) were coated with soluble VCAM-1 at 10 µg/ml in the presence of a 2 µg/ml HSA carrier. The plates were washed three times with PBS and blocked with HSA (20 µg/ml in PBS) for 2 h at RT. Alternatively, washed plates were coated with 10 µg/ml of the CCL2, CCL5, CCL11, CXCL8, CXCL9, MIG CXCL10, CXCL11, and CXCL12 chemokine in PBS for 30 min at RT before being blocked with HSA. The plates were assembled as the lower wall of a parallel wall flow chamber and mounted on the stage of an inverted microscope. The peptide, as described previously (10 µg/ml), was allowed to settle on the substrate-coated chamber wall for 10 min at 37°C and was then washed. T cells (5 × 10^6^/ml, purity >98%) were suspended in binding buffer, perfused into the chamber and allowed to settle on the substrate-coated chamber wall for 1 min at 37°C. Flow was initiated and increased in 2- to 2.5-fold increments every 5 s, generating controlled shear stress on the wall. Cells were visualized using the 20× objective of an inverted phase-contrast Diaphot microscope (Nikon, Japan) and photographed with a long integration LIS-700 CCD video camera (Applitech, Holon, Israel) connected to a video recorder (AG-6730 S-VHS, Panasonic, Japan). The number of adherent cells resisting detachment by the elevated shear forces was determined after each interval by analyzing the videotaped cell images and was expressed as the percentage of originally settled cells. All the adhesion experiments were performed at least three times in multiple test fields.

#### ELISA for the Binding of BKT130 to Different Chemokines

ELISA assay was performed in order to evaluate the binding of BKT130Fc to different chemokines. Plates were pre-coated with different chemokines at concentration of 150 ng/well/100 μl in coating buffer (0.1 M Carbonate pH 9.5) and incubated over night at 4°C. Plates were than washed once with washing buffer (PBS with 0.05% Tween) and blocked with 200 µl of blocking buffer (4% BSA in PBS). Following 1 h of incubation at RT the plates were washed once with washing buffer and 100 µl of BKT130 (0, 1, 10, and 50 µg/ml) were added into each well. Following 2 h of incubation at RT plates were washed five times with washing buffer and second antibody (Goat anti-Human IgG HRP conjugate) was added at dilution of 1:1000 for 2 h at RT. Following incubation plates were washed five times with washing buffer and 3,3′-5,5′-tetramethylbenzidine (TMB) substrate was added (100 µl/well). 30 min later the reaction was stop with 50 µl of stop solution (1 M H_2_SO4) and the absorbance was measured by ELISA Reader at wavelength of 450 nm.

#### BiAcore Analysis of Peptibodies

The binding of the peptibodies (BKT130Fc and BKT120Fc) to the chemokines CXCL11, CXCL10, CXCL9, CCL2, CCL11, and CCL5 was done using BiAcore analysis.

Biomolecular Interaction Analysis using surface plasmon resonance (SRP) was performed.

To immobilize the chemokines to the CM5 sensor chip (BIAcore), the carboxyl groups on the matrix were activated by adding 10 µl of 1:1 mixture of succinimide (NHS) and carbodiimide (EDC) to form active esters which react spontaneously with amine groups on the chemokines (BIAcore kit, according to the manufacturer orders). Then the chemokines were immobilized by using standard amine-coupling chemistry to a level of ~1,000 response units (RU; 1000 pg/mm^2^) onto a CM5 sensor chip using a BIAcore 3000 biosensor.

In order to test the interaction of the peptibodies to the chemokines, serial dilutions of the peptibodies, 1, 0.5, 0.25, 0.125, and 0.032 mM were tested by the BIAcore analysis at a flow rate of 10 µl/min. Real-time binding data of the peptibodies to the different chemokines immobilized were plotted as the mass of peptide binding to immobilized chemokines in RU as a function of time. Data were globally analyzed with the analysis software BIAEVALUATION 3.0 (BIAcore) using a 1:1 mass transport mode. The equilibrium dissociation constant was calculated using the relationship between the mean values for Kon and Koff (K_D_ = Koff/Kon). To demonstrate reproducibility, sensorgrams of four concentrations of the peptide in question were overlaid.

#### *In Vitro* Migration Assay

The migration of cells *in vitro* toward the various chemokines was assessed using transmigration plates that were 6.5-mm in diameter and 5-µm in pore size (Costar). Migration toward CCL2 (10 ng/ml) and CCL5 (20 ng/ml) was assessed using THP-1 cell monocytes (ATCC cat# TIB202), while migration toward CXCL12 (100 ng/ml) was assessed using freshly isolated human CD4+ T cells, and migration toward CXC10 (500 ng/ml) and CXCL11 (500 ng/ml) was assessed using Jurkat cells overexpressing CXCR3. Briefly, 600 µl of RPMI supplemented with 1% FCS was added to the lower chamber of transwells supplemented with the different chemokines. The chemokines were incubated with BKT130Fc, BKT120Fc, or BKT110Fc (10 µg/ml) for 30 min prior to the initiation of the migration assay. After 30 min of incubation, 2 × 10^5^ cells/well were added to the upper chambers of the transmigration plates in a total volume of 100 μl of RPMI plus 1% FCS. Cells that migrated to the bottom chamber of the transwell within 3 h were counted using FACScalibur.

When Jurkat-CXCR3 cells were used, the upper wells were pre-coated with fibronectin (10 µg/ml) for 1 h at 37°C prior to the migration assay.

CD4+ T cells were obtained from fresh whole blood using a RosetteSep Human CD4+T Cell Enrichment Cocktail (STEMCELL Technologies) according to the manufacturer’s instructions.

### *In Vivo* Assay

#### Animals

C57BL/6, SJL, and BALB/c mice (7–10 weeks old) were purchased from Harlan (Rehovot, Israel) and maintained under specific pathogen-free conditions at the Hebrew University Animal Facility (Jerusalem, Israel). All studies were carried out in accordance with the recommendations of Hebrew University Animal Facility (Jerusalem, Israel). The protocols were approved by the Animal Care and Use Committee of Hebrew University. The protocol numbers of the animal ethics committee that were used in this study are: MD-08-11724-2, MD-13-13659-3, MD-08-11460-4, MD-11-12355-5, and MD-09-12250-4.

#### Pharmacokinetics (PK) in Mice

Pharmacokinetic analysis of the peptibodies in mice was performed in C57BL/6 mice. First the mice were intravenously (i.v.) injected with different doses of BKT130Fc (25, 50, and 100 μg/mouse) The mice were bled for 2 h, 24 h, and 6 days post-injection, and serum was extracted. The level of peptibodies in their serum was tested using a Human Total IgG ELISA kit (ICL) according to the manufacturer’s instructions.

Next the mice were i.v. injected with 50μg/mouse of BKT130Fc or BKT120Fc. The mice were bled for 2 h, 24 h, or 6, 11, and 18 days post-injection, and the level of peptibodies in their serum was tested using a Human Total IgG ELISA kit.

#### Production of Antibodies against BKT130Fc

C57BL/6 mice were i.v. injected with 50 μg of BKT130Fc once or twice a week for total of four injections. One-week after the last injection serum was extract and ELISA was performed.

ELISA assay was performed in order to evaluate the level of antibodies against BKT130Fc or BKT120Fc in the mice serum. Plates were pre-coated with either BKT130Fc, BKT120Fc or BSA at concentration of 150 ng/well/100 μl in coating buffer (0.1 M Carbonate pH 9.5) and incubated over night at 4°C. ELISA was done as described at section 2.12.2.

#### Delayed-Type Hypersensitivity (DTH)

Female BALB/c mice were sensitized on their shaved abdominal skin with 100 µl of 2% oxazalone dissolved in acetone/olive oil [4:1 (vol/vol)] applied topically (day 0). DTH sensitivity was elicited 6 days later by challenging the mice with 20 µl of 0.5% oxazalone in acetone/olive oil, with 10 µl being administered topically to each side of their right ears. BKT130Fc, BKT120Fc, or BKT110Fc were intravenously injected (50 µg/mouse) on day 0 and 24 h before the challenge (day 5) (total of 100 µg/mouse). Control group mice were subcutaneously injected with dexamethasone (100 µg/mouse in a total volume of 200 µl). Right ear swelling was measured 24 h after the challenge using a micrometer digital caliper (Mitutoyo Corp, Tokyo, Japan). Swelling of the left ear served as the control.

#### Antigen-Induced Arthritis (AIA)

Antigen-induced arthritis was induced as previously described by Coelho et al. (17). Briefly, the mice (8- to 10-week-old male C57BL/6 mice) were immunized intradermally at the base of their tail with 500 µg of mBSA (Sigma) in 100 µl of a saline emulsion and an equal volume of CFA (Difco Detroit, MI, USA) on day 0. Twelve days later, the mice were challenged with antigens. Each mouse received an injection of 10 µg of mBSA in 10 µl of sterile saline in their left knee joint (17, 18). In some mice, BKT130Fc or BKT120Fc (50 µg/mouse) were i.v. injected 24 h before the challenge. Naïve mice challenged with sham PBS served as the controls. Mice were killed 24 h after the antigen challenge.

The mouse knee cavities were washed with 3% BSA in PBS. The number of infiltrated cells was evaluated using FACScaliber. Periarticular tissue was removed from their joints to evaluate myeloperoxidase (MPO) activity.

For histology, the other knee joint form each mouse was removed and fixed with 8% paraformaldehyde for 12 h. The joints were then incubated in 20% EDTA at pH 7.2 for 6 days to decalcify the bones. Samples were washed with PBS and dehydrated. After being embedded in paraffin, the joints were sliced into 6-µm-thick sections and stained with hematoxylin and eosin (H&E) (17, 19).

The extent of neutrophil accumulation in the mouse tissues was measured by assaying MPO activity as described (20, 21). Briefly, the knee joint was removed and frozen at −20°C. Upon thawing of the sample, the tissue (0.1 gm of tissue per 1.9 ml of buffer) was homogenized and processed for the determination of MPO activity. The assay included 3,3′-5,5′ TMB (Sigma) in PBS as the color reagent.

#### Experimental Autoimmune Encephalomyelitis (EAE)

Experimental autoimmune encephalomyelitis was induced in female SJL and C57BL/6 mice. Mice were immunized subcutaneously in their flanks (day 0) with 100 µg of PLP139-151 or 250 µg MOG35-55, respectively. Proteolipid protein (PLP) and myelin oligodendrocyte glycoprotein (MOG) peptides were emulsified in an equal volume of CFA containing 800 µg of mycobacterium tuberculosis H37RA (Difco). Mice were injected intraperitoneally with 300 ng of pertussis toxin (PTX) on days 0 and 2.

Mice were i.v. injected once with either BKT130Fc, BKT120Fc, or BKT110Fc (50 µg/mouse) on day 9 post-immunization. Spinal cord tissue samples were collected 30 days after the induction of disease, fixed in 4% paraformaldehyde, dehydrated and embedded in paraffin. Spinal cord sections were cut at 6 µm and processed for histological analysis by H&E staining to evaluate immune cell infiltration and by luxol fast blue to mark the area of demyelination.

Blood samples were collected on day 30 post-immunization, and serum was extracted for cytokine analysis. Cytokine levels were evaluated using a Mouse Th1/Th2 Cytokine kit/BD cytometric bead array according to the manufacturer’s instructions.

Clinical assessment of EAE was performed according to the following criteria: 0, no disease; 1, decreased tail tone; 2, hind limb weakness or partial paralysis; 3, complete hind limb paralysis; 4, front and hind limb paralysis; 5, moribund state. For all EAE experiments, there were 10 to 12 mice per group.

### Statistical Analysis

Data are presented as the mean ± SD. Statistical comparisons of the means were performed using two-tailed unpaired Student’s *t*-tests. Differences of *p* ≤ 0.05 were considered statistically significant.

## Results

### Identification of CBPs that Inhibited the Chemokine-Induced Adhesion and Migration of Immune Cells

Phage display libraries were screened for their ability to bind five immobilized human chemokines CCL11, CXCL9, CXCL8, CXCL12, and CCL2 as described in the Section “Materials and Methods.” Following three to four cycles of panning 156 different phages were identified (Table S1 in Supplementary Material). Sixty-nine different phages were further selected for their ability to bind CCL2 using ELISA assay (Table S2 in Supplementary Material). Whereas most of the CCL2-binding phages appeared in only one bacterial colony following three cycles of enrichment (*n* = 65), the phage carrying the BKT130 peptide appeared in 12 different colonies, suggesting a significant enrichment for this CCL2-binding phage throughout the process (Table S2 in Supplementary Material).

**Figure 1:**
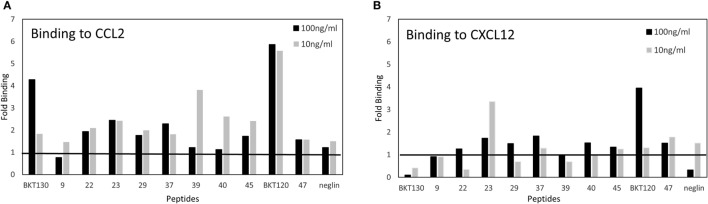
ELISA of chemokine-binding peptides (CBPs) with CCL2 and CXCL12. ELISA was performed on biotinylated peptides to detect their interaction with **(A)** CCL2 or **(B)** CXCL12. The data are presented as the fold binding that was obtained using two concentrations of the various peptides (10 and 100 ng/ml).

**Figure 2:**
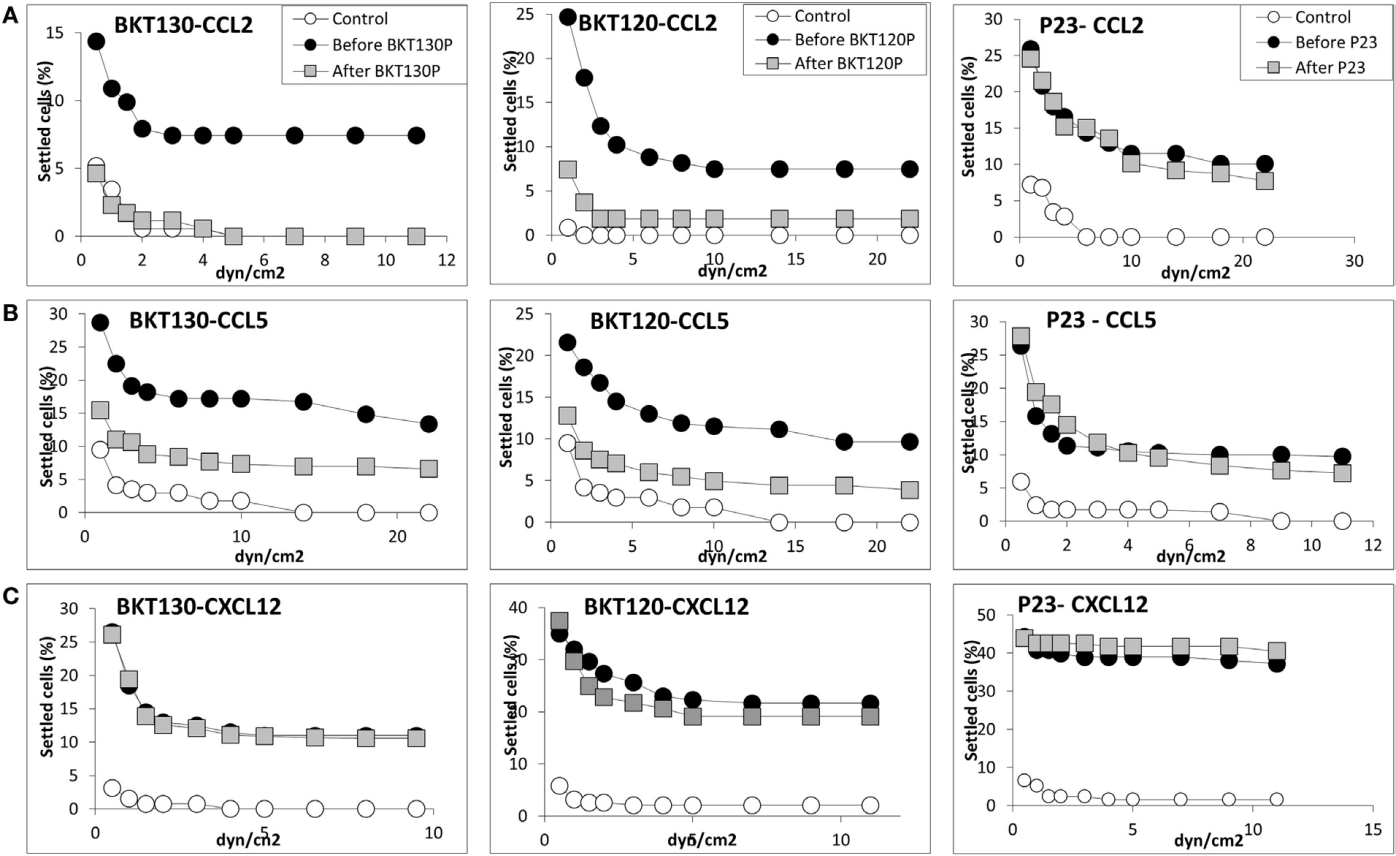
Effect of various peptides on the chemokine-induced immune cell-dependent adhesion to VCAM-1. Adhesion was measured using the laminar flow assay. The number of adherent cells resisting detachment by elevated shear forces (dyn/cm^2^) is expressed as the percentage of originally settled cells. The effects of the peptides BKT130, BKT120, and peptide 23 (P23) on the **(A)** CCL2-, **(B)** CCL5-, and **(C)**CXCL12-induced immune cell-dependent adhesion to VCAM-1 were measured. All the adhesion experiments were performed at least three times on multiple test fields.

Next, we synthesized biotinylated peptides and analyzed their ability to bind CCL2. We found that BKT120, BKT130, and other peptides, interacted with CCL2 and, to a lesser extent, with CXCL12 (Figure 1). The ability of BKT120, BKT130, and peptide 23 [P23 that was fished from the library by another chemokine (CCL11)] to prohibit CCL2 (Figure 2A), CCL5 (Figure 2B), and CXCL12 (Figure 2C) from inducing immune cell-dependent adhesion to the endothelial adhesion molecule VCAM-1 was tested. BTK120 and BTK130 inhibit CCL2- and CCL5-mediated adhesion to VCAM-1 but have not effect on adhesion mediated by CXCL12 (Figure 2). Peptide 23 (P23), which was used as the control, did not affect the CCL2-, CCL5-, or CXCL12-dependent adhesion of T cells to VCAM-1.

### Turning CBPs into Peptibodies Enhanced Their Biological Activity

**Figure 3:**
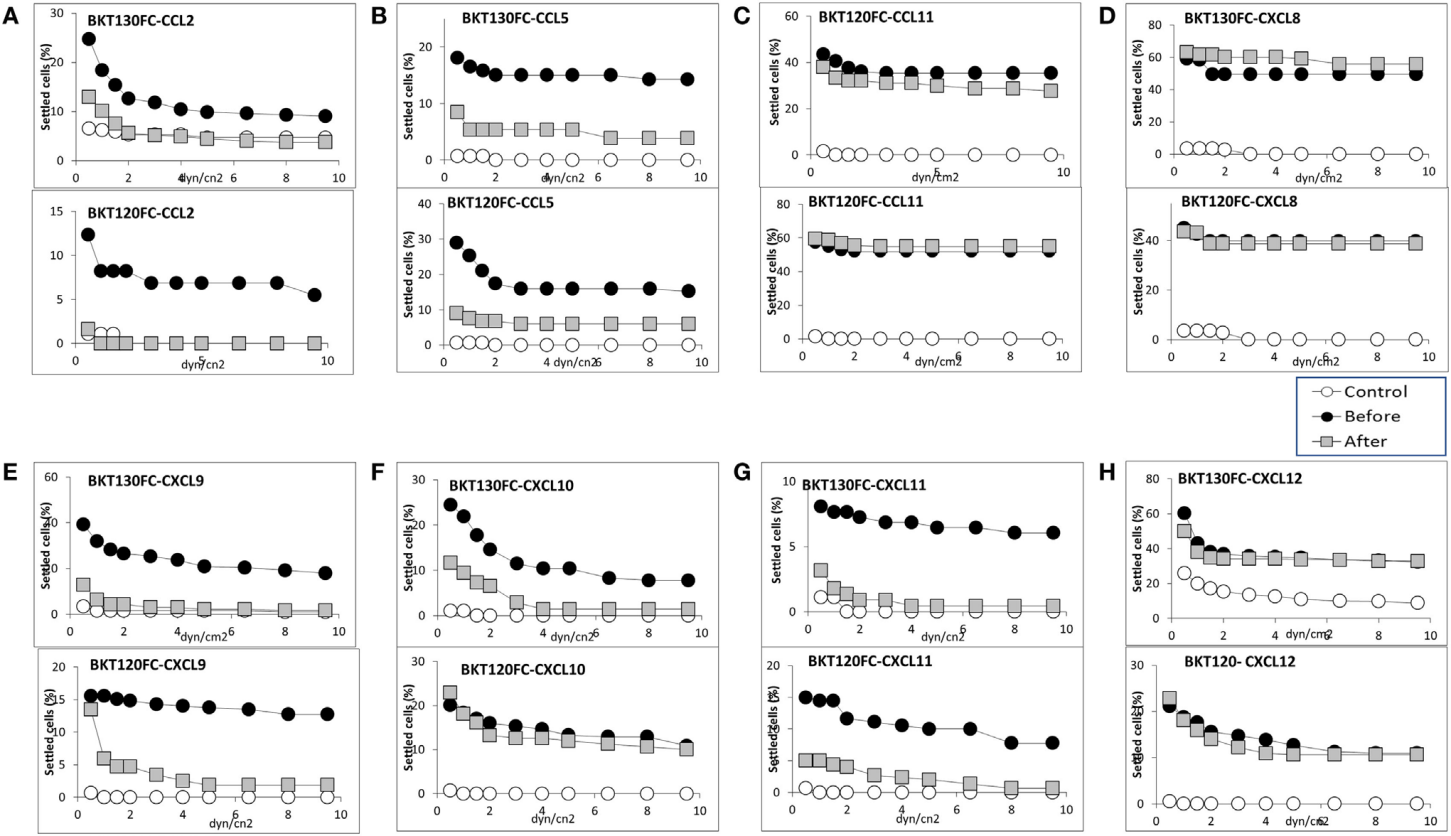
Effect of the peptibodies BKT130Fc and BKT120Fc on the chemokine-induced immune cell-dependent adhesion to VCAM-1. Adhesion was measured using the laminar flow assay. The number of adherent cells resisting detachment by elevated shear forces (dyn/cm^2^) is expressed as the percentage of originally settled cells. The effects of the peptibodies BKT130Fc and BKT120Fc on the **(A)** CCL2-, **(B)** CCL5-, **(C)** CCL11-, **(D)** CXCL8-, **(E)** CXCL9-, **(F)** CXCL10-, **(G)** CXCL11-, and **(H)** CXCL12-induced immune cell-dependent adhesion to VCAM-1 were measured. All the adhesion experiments were performed at least three times on multiple test fields.

Two of the CCL2-binding peptides (BKT120, BKT130) as well as control peptide (BKT110) were cloned into Fc-peptides (peptibodies). The BKT120, BKT130, and BKT110 peptibodies are composed of two moieties, a biologically active peptide and an Fc region. The effect of BKT120Fc and BKT130Fc on the ability of (Figure 3A), CCL5 (Figure 3B), CCL11 (Figure 3C), CXCL8 (Figure 3D), CXCL9 (Figure 3E) CCL2, CXCL10 (Figure 3F), CXCL11 (Figure 3G), and CXCL12 (Figure 3H) to induce immune cell-dependent adhesion to the endothelial adhesion molecule VCAM-1 was tested. BKT120Fc and BKT130Fc both inhibited the function of CCL2, CCL5, CXCL9, and CXCL11 but did not inhibit the function of CXCL8, CXCL12, and CCL11. BKT130Fc, but not BKT120Fc, inhibited CXCL10 function (Figure 3F). BKT110Fc did not inhibit the function of CCL2, CCL11, CXCL8, and CXCL10 (Figures S1A–D in Supplementary Material).

The effect of BKT110Fc, BKT120Fc, and BKT130Fc on the ability of CCL2, CCL5, CXCL10, CXCL11, and CXCL12 to induce the migration of immune cells was studied in more detail. BKT130Fc (10 µg/ml) was more effective then BKT120Fc in inhibiting the migration of immune cells in response to CCL2, CCL5, CXCL10, and CXCL11 but did not inhibit the migration of cells in response to CXCL12. BKT110 had no effect on the migration toward CCL2, CCL5, CXCL10, and CXCL11 whereas it has significant inhibition on the migration of cells in response to CXCL12 (Figures 5A–E).

### Binding of BKT130Fc and BKT120Fc to Different Chemokines

In order to analyze the binding characteristics of the interaction between BKT130Fc/BKT120Fc and the chemokines CXCL11, CXCL10, CXCL9, CCL2, CCL11, and CCL5, a BiaCore assay was performed (Table 1). The affinity of BKT130Fc to CXCL11, CXCL10 and CCL5 was the highest with K_D_ of 8.3 E−0.8, 3.8 E−0.8, and 1.8 E−0.8, respectively. The K_D_ for CXCL9 binding was 1.3 E−0.7 and lower affinity binding to CCL2 and CCL11 with K_D_ of 6.5 E−0.6 and 2.7 E−0.5 were observed. The affinity of BKT120Fc to CXCL10, CCL2, and CXCL9 was the highest with K_D_ of 6.3 E−0.9, 4.6 E−0.8, and 1.8 E−0.8, respectively. The K_D_ for CXCL11 and CCL11 was lower with KD of 3.7 E−0.7 and 5 E−0.6, respectively (Table 1).

**Table 1:** BiAcore K_D_ Values for the interactions of BKT130Fc and BKT120Fc with various chemokines.

	BKT130Fc		BKT120Fc	
	BKT130Fc (K_D_)	Chi-squared (χ^2^)	BKT120Fc (K_D_)	Chi-squared (χ^2^)
CXCL11	8.3 E−0.8	0.51	3.7 E−0.7	0.76
CXCL10	3.8 E−0.8	1.05	6.3 E−0.9	0.99
CXCL9	1.3 E−0.7	1.05	1.8 E−0.8	0.61
CCL2	6.5 E−0.6	0.65	4.6 E−0.8	0.75
CCL11	2.7 E−0.5	1.2	5 E−0.6	0.51
CCL5	1.8 E−0.8	0.6		

**Figure 4:**
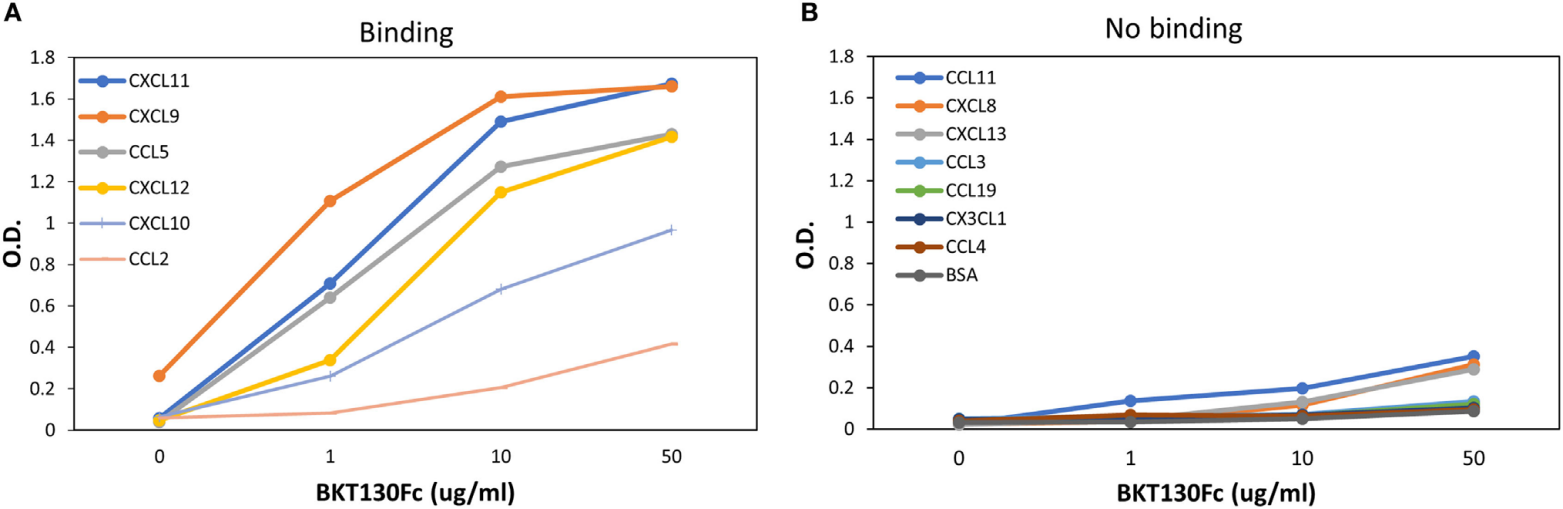
ELISA for the binding of BKT130Fc to different chemokines. ELISA was performed with several dilutions of BKT130Fc (1, 10, and 50 μg/ml) to detect the interaction to different chemokines that were bound to the plates. The data are presented as the optical density (O.D.) obtained at wavelength of 450 nm. **(A)** presents the chemokines that demonstrate binding to BKT130Fc and **(B)** presents the chemokine with no binding to BKT130Fc.

ELISA assay was developed to further study the binding of BKT130Fc to different chemokines (Figure 4). BKT130Fc demonstrated a strong binding to CXCL11, CXCL9, CCL5, and CXCL12 and lower binding to CXCL11 and CCL2. No binding of BKT130Fc was found to CCL11, CXCL8, CXCL13, CCL3, CCL19, CX3CL1, and CCL4.

**Figure 5:**
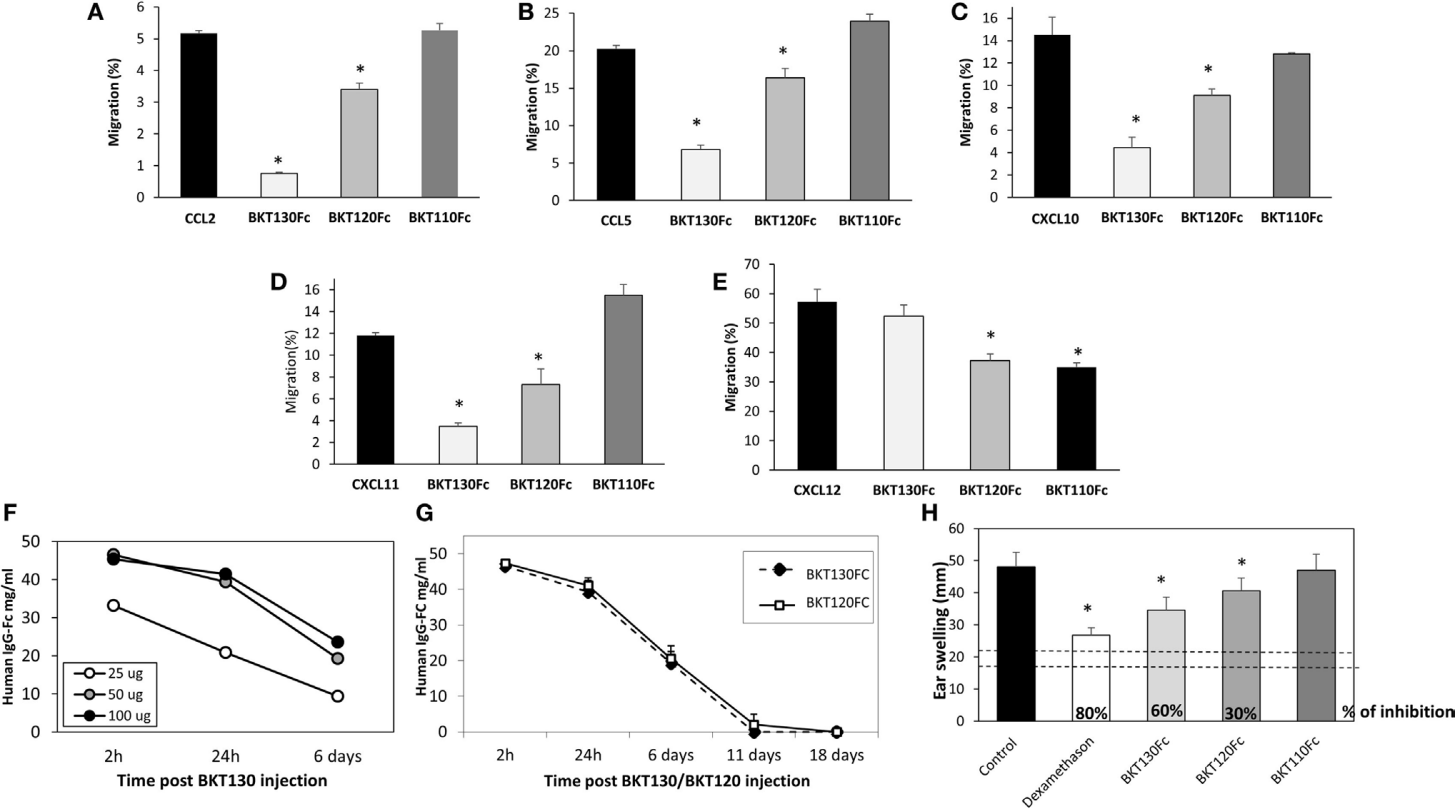
The peptibodies BKT130Fc and BKT120Fc inhibit the *in vitro* migration of different chemokines. A transmigration assay was performed to study the effects of the BKT130Fc, BKT120Fc and BKT110Fc peptibodies on *in vitro* migration. **(A)** Migration toward CCL2 was assessed using THP-1 cells. **(B)** Migration toward CCL5 was assessed using THP-1 cells. **(C)** Migration toward CXCL10 was assessed using Jurkat cells overexpressing CXCR3. **(D)** Migration toward CXCL11 was assessed using Jurkat cells overexpressing CXCR3. **(E)** Migration toward CXCL12 was assessed using CD4+ T cells. BKT130Fc, BKT120Fc, and BKT110Fc were added at 10 µg/ml. The results, analyzed using Student’s *t*-tests (*p ≤ 0.05), are expressed as the mean percentage of migration ±SD. **(F)** Dosing of BKT130Fc pharmacokinetics (PK). C57BL/6 mice (*n* = 3) were intravenously (i.v.) injected with 25, 50, or 100 μg of BKT130Fc. The serum peptibody levels were tested after 2 h, 24 h, and 6 days. **(G)** PK of BKT130Fc and BKT120c in mice. C57BL/6 mice (*n* = 5) were i.v. injected with BKT120Fc or BKT130Fc at 50 µg/mouse. The serum peptibody levels were tested after 2 h, 24 h, and 6, 11, and 18 days. **(H)** BKT130Fc and BKT120Fc inhibited delayed-type hypersensitivity (DTH) *in vivo*. DTH model was established in female BALB/c mice (*n* = 12). BKT120Fc, BKT130Fc, or BKT110Fc were intravenously injected (50 μg/mouse) on day 0 and 24 h before the challenge (day 5) (total of 100 μg/mouse). Dexamethasone (100 µg/muse) served as the positive control. The two dotted lines indicate the normal range of ear swelling. The results, analyzed using Student’s *t*-tests (*p ≤ 0.05), are expressed as the mean ± SE of ear swelling (mm). All the DTH experiments were performed at least four times.

The results obtained from the BiaCore and ELISA assays correlate with the ability of the peptibodies to inhibit the function of CCL5, CXCL9, CXCL10, CXCL11, and to lesser extent CCL2 (Figures 3 and 5). BKT130Fc/BKT120Fc did not bind or inhibit the function of CCL11 and CXCL8 (Figures 3C,D). BKT130Fc did not inhibit the migration and adhesion of immune cells in response to CXCL12 (Figures 3H and 5E). However, BKT130Fc strongly bind CXCL12 suggesting that binding to chemokines does not always inhibit their function (Figure 4A). Similar results were obtained for BKT120 peptide (Figure 1) which interacts with CXCL12 in ELISA assay but do not inhibit its function (Figure 2).

### PK of BKT130Fc and BKT120Fc in Mice

To evaluate the PK of BKT130Fc in mouse sera the peptibody was injected i.v once at three different doses of 25, 50, and 100 μg/mouse. The level of BKT130Fc in the sera was tested following 2h, 24h, and 6 days. The best PK for BKT130Fc was observed following injection of 50 and 100 μg of BKT130Fc. Injection of 25 μg/mouse gave a significant less effective PK for BKT130Fc (Figure 5F). Therefore, we choose to use the 50 μg/mouse of BKT130Fc throughout all the *in vivo* experiments. When 50 μg of peptibodies were injected into mice, BKT120Fc and BKT130Fc retained certain desirable antibody features, including an increased apparent affinity through the avidity conferred by the dimerization of two Fcs and a prolonged PK, of IC50 = 6 days (Figure 5G).

### Production of Antibodies against BKT130Fc

In order to check the immunogenicity of BKT130Fc, mice were i.v injected with BKT130Fc once or four times. Antibodies against BKT130Fc were not produced following single injection of 50 μg of BK130Fc. The level of antibodies against BKT130Fc was increased following four injections (Figure S2A in Supplementary Material). Interestingly, these antibodies could not recognize BKT120Fc (Figure S2B in Supplementary Material). These results suggest that the antibodies produced recognize the BKT130 peptide and not the Fc portion.

### BKT130Fc and BKT120Fc Inhibited DTH

Delayed-type hypersensitivity is a process immunologically similar to cell-mediated immunity, and the inflammatory DTH reaction is mediated by effector TH1/TH17 memory T lymphocytes (22). In this *in vivo* assay, lymphocytes infiltrate to the injection site (ear) of an antigen against which the immune system has been primed. The inflammatory reaction is characterized by the redness and swelling of the ear following antigenic challenge. The chemokines CXCL10, CXCL9, CCL3, and CCL5 were shown to regulate this inflammatory process (23, 24). The ability of BKT110, BKT120Fc and BKT130Fc, i.v. administered at 50 µg/per a mouse once, on days 1 and 6, to inhibit the DTH response in mice 24 h before the second sensitization was tested. Dexamethasone, BKT130Fc, and BKT120Fc significantly inhibited the inflammatory process (80, 60, and 30%, respectively) (Figure 5H). Importantly, BKT110 had no effect on DTH. Overall, our results suggest that BKT130Fc has a more potent inhibitory effect than BKT120Fc on inflammatory chemokines *in vitro* and *in vivo*.

### BKT130Fc and BKT120Fc Inhibited the Infiltration of Inflammatory Cells into the Synovial Cavity in an Antigen-Induced Mouse Arthritis Model

**Figure 6:**
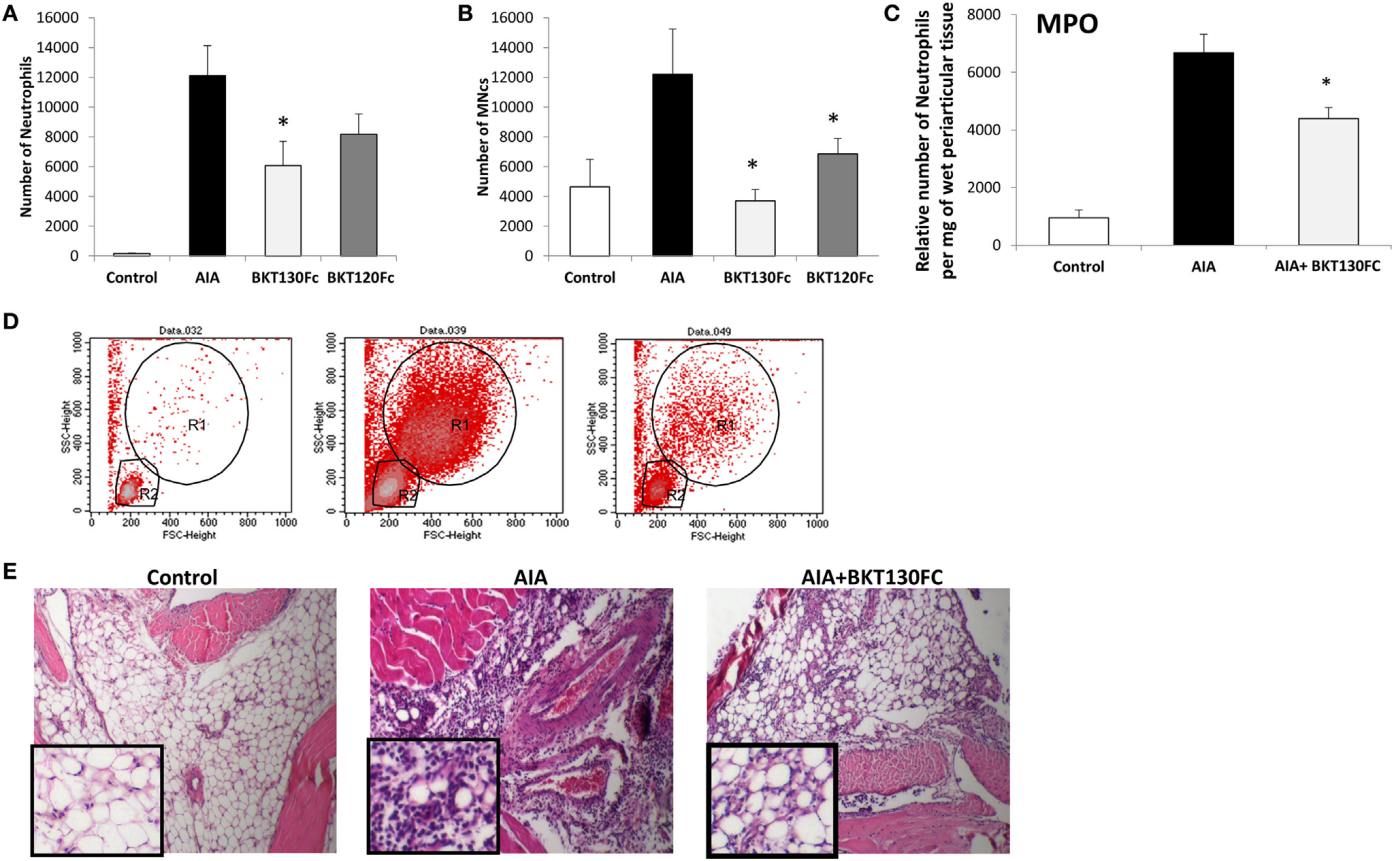
BKT130Fc and BKT120Fc inhibited the infiltration of inflammatory cells into the synovial cavities of mice of the antigen-induced arthritis (AIA) model. Male C57BL/6 mice (*n* = 12) were immunized with methylated bovine serum albumin. BKT130Fc or BKT120Fc were administered *via* i.v. once at 50 µg/per a mouse, 24 h before the challenge. The control group represents normal mice, the AIA group represents untreated AIA mice and the BKT130Fc or BKT120Fc groups represent AIA mice treated with BKT130Fc or BKT120Fc, respectively. The number of infiltrated cells into the knee cavity was evaluated by FACScaliber: **(A)** number of neutrophils, **(B)** number of MNCs. Representative FACS analysis of cells within the knee cavity is presented in **(D)**. The R1 gate shows the neutrophil population and the R2 gate shows the MNCs. The level of myeloperoxidase (MPO) activity is presented in **(C)** as the relative number of neutrophils per mg of wet periarticular tissue. **(E)** Histology analysis of the knee joint shows the infiltration of inflammatory cells following hematoxylin and eosin (H&E) staining.

The effect of BKT130Fc and BKT120Fc on RA was studied using an AIA model. BKT130Fc or BKT120Fc were i.v. administered once, at 50 µg/per a mouse 24 h before the challenge. BKT130Fc partially inhibited the infiltration of neutrophils into the synovial cavity, while it completely abrogated the infiltration of MNCs (including monocytes and lymphocytes) (Figure 6). BKT120Fc had less significant effect compare to BKT130Fc.

### BKT130Fc and BKT120Fc Reduced the Severity of EAE by Inhibiting the Infiltration of Immune Cells into the CNS

The best animal model for MS is an EAE model, which can be induced by immunization using antigens derived from myelin, such as PLP fragments and MOGs.

**Figure 7:**
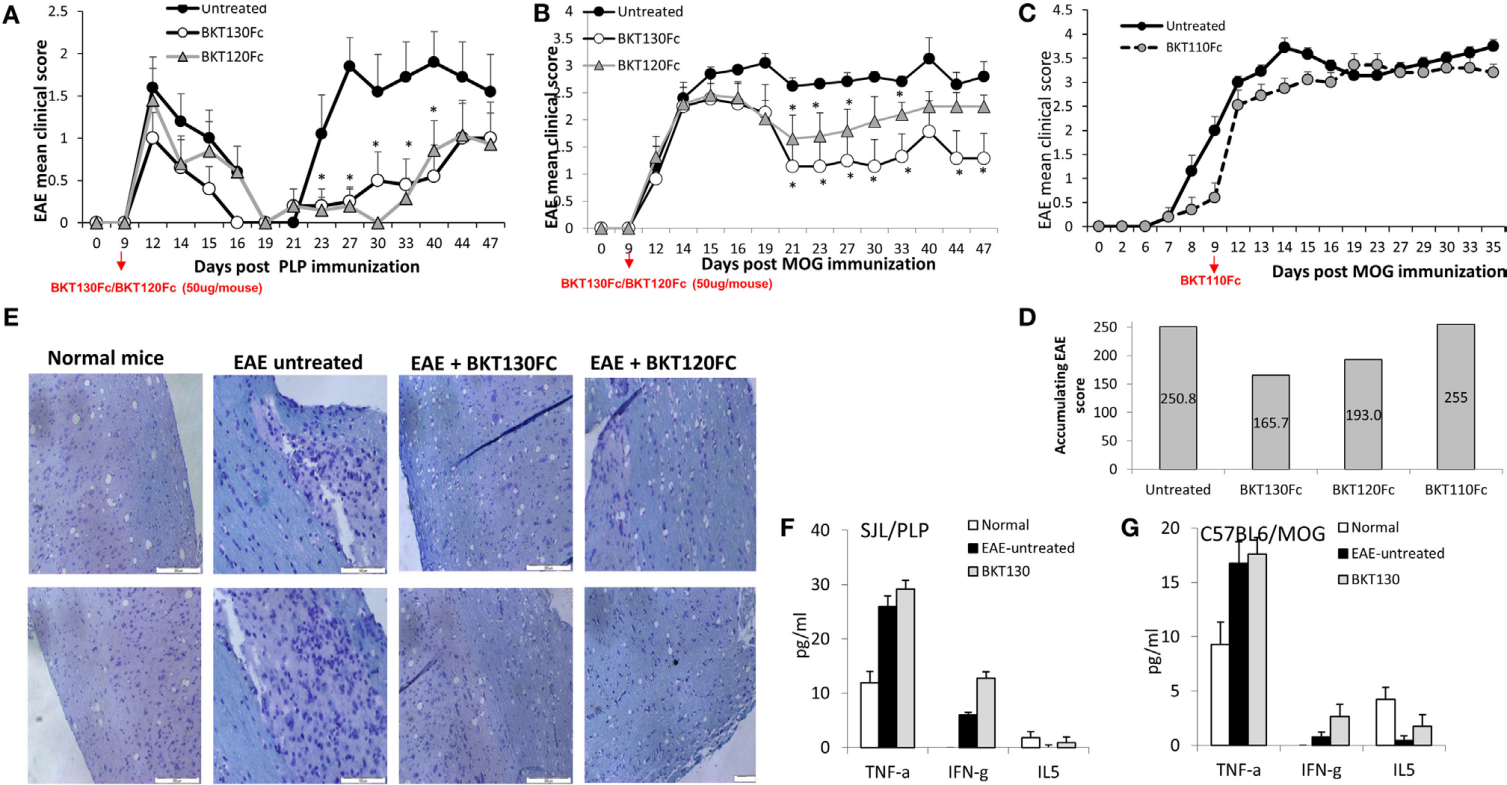
BKT130Fc and BKT120Fc reduced the severity of experimental autoimmune encephalomyelitis (EAE) by inhibiting the infiltration of immune cells into the central nervous system (CNS). **(A)** Female SJL mice (*n* = 12) were immunized with the proteolipid protein (PLP) peptide to induce relapsing-remitting EAE. BKT130Fc or BKT120Fc were intravenously administered once (50 μg/mouse) on day 9 post-disease induction. **(B)** Female C57BL/6 mice (*n* = 12) were immunized with a myelin oligodendrocyte glycoprotein (MOG) peptide to induce chronic EAE. BKT130Fc or BKT120Fc were intravenously administered once (50 μg/mouse) on day 9 post-disease induction. The data are presented as the EAE mean clinical score measured for 47 days post-immunization. **(C)** Female C57BL/6 mice (*n* = 8) were immunized with a MOG peptide to induce chronic EAE. BKT110Fc was intravenously administered once (50 μg/mouse) on day 9 post-disease induction. **(D)** Accumulating score of MOG induced EAE after treating with BKT130Fc, BKT120Fc, or BKT110Fc. **(E)** Histology analysis of the spinal cords from SJL mice shows the infiltration of immune cells into the CNS following hematoxylin and eosin (H&E) staining and the area of demyelination marked with luxol fast blue (light blue staining = demyelination area). **(F–G)** Cytokine levels in the sera of mice on day 30 post-immunization as measured by the Mouse Th1/Th2 Cytokine Kit Array in **(F)** SJL mice immunized with PLP and **(G)** C57BL/6 mice immunized with MOG. The results, analyzed using Student’s t-tests (*p ≤ 0.05), are expressed as the mean ± SE. All the EAE experiments were performed at least three times.

The effect of BKT130Fc and BKT120Fc on EAE was studied in two different models. The PLP induced relapsing-remitting EAE and the MOG induced chronic EAE (25). BKT130Fc or BKT120Fc were intravenously administered once (50 μg/mouse) on day 9 after disease induction. In the SJL/PLP model, both BKT130Fc and BKT120Fc significantly prolonged the remission time and reduced the severity of the second relapse (Figure 7A), with an observed accumulating clinical score of 159 in the control group and an observed accumulating clinical score of 62 and 63 following BKT130Fc and BKT120Fc treatment, respectively. In the second relapse (starting from day 27), clinical signs appeared in 70–80% of the control mice compared to only 20% of the mice treated with BKT130Fc or BKT120Fc. This effect was accompanied by a lack of weight loss in the treated animals (data not shown). Histology of the spinal cord sections demonstrated that treatment with BKT130Fc or BKT120Fc inhibited the infiltration of immune cells into the CNS while also reducing the demyelination area (Figure 7E). Interestingly, BKT130Fc did not affect the peripheral balance between the Th1 and Th2 cytokine levels (Figure 7F).

Similar to the effect in the relapsing-remitting EAE model, a single i.v. injection of BKT130Fc (50 μg/mouse on day 9) significantly inhibited disease severity in the chronic EAE model (Figure 7B). BKT120Fc had less significant effect compare to BKT130Fc.

The control mice demonstrated an accumulating clinical score of 250.8 compared to 165.7 in mice treated with BKT130Fc and 193 following treatment with BKT120Fc. Importantly, BKT110 had no effect on MOG induced EAE with accumulating clinical score of 255 (Figures 7C,D).

The effect of BKT130Fc was accompanied by a lack of weight loss in the treated animals (data not shown). Like the first model, BKT130Fc did not affect the peripheral levels of Th1/Th2 cytokines (Figure 7G).

## Discussion

Effective inflammatory responses involve chemokine-dependent homing, retention, and the resolution of immune cell trafficking to damaged tissues (3). In addition to their physiological roles, chemokines and their receptors play a key role in the pathogenesis of autoimmune diseases, inflammation, viral infection, and cancer (3). However, except for selective CCR5 antagonists for HIV (Maraviroc) and a stem cell mobilizer (Mozobil), the promise of obtaining new therapeutics related to blocking chemokine receptor function in autoimmune diseases, inflammation, and cancer has not yet been realized.

In the past 15 years, several phase II/III clinical trials with antagonists against the chemokine receptors CCR1, CCR2, CCR3, CCR5, CCR9, CXCR1, CXCR2, and CXCR3 have failed to have significant clinical benefits in patients with autoimmune and inflammatory diseases, such as RA, multiple sclerosis (MS), IBD, psoriasis, and asthma (4, 5).

Reasons for such failures may be the lack of animal model predictability, relevance of the target to the human disease, diversity in the molecular and genetic drives of the disease within patient population, poor drug-like properties of small-molecule antagonists, ineffective dosing, target disease redundancy, and the use of multiple chemokine receptors (4, 5).

As quoted in the paper by Horuk et al. (5) CCR1, CCR2, CCR5, CCR6, and CXCR3 have all been implicated in the pathophysiology of MS. Moreover, MS is an extremely heterogeneous disease that consists of at least four distinct patterns of demyelination. Patients with MS that are enrolled in clinical trials could feasibly have varying pathophysiological subtypes that are perhaps driven by different chemokines and chemokine receptors. Multiple chemokine receptors, including CCR1, CCR2, CCR5, CXCR2, and CXCR3, seem to have roles in RA. Therefore, there is a strong possibility that receptor redundancy could account for the failure of CCR1 and CCR2 antagonists in RA clinical trials (4, 5).

In an elegant paper by Schall and Proudfoot the authors suggested that the chemokine/chemokine receptor system works as a network of signals in which redundancy is rare (26). They suggested that chemokine expression and chemokine receptor usage by immune cells is regulated in a cell type, time, and tissue-specific manner that is very specific and those not support the idea of redundancy in the system. It was, therefore, suggested that reasons for clinical failures is imbedded in the *in vivo* potency of the drug tested; in sufficient bioavailability, in sufficient metabolic stability and toxicity that prevented achieving high levels of free drug in the plasma. In addition, it was suggested that our miss understanding of the multidimensional nature of the inflammatory diseases, prevent us from effectively selecting the target and time of the intervention. It was, therefore, hypothesized that by deepen our understanding of the chemokine/chemokine receptor network in disease and improving our drugs a successful chemokine-targeted drugs for inflammatory diseases is visible.

Binding to multiple chemokines as a strategy for inhibiting inflammation is a phenomenon used by hosts as well as by different pathogens to reduce inflammation and immunity. Therefore, the inhibition of single chemokine receptors as an anti-inflammatory strategy most likely did not prevail during the evolution of hosts and pathogens.

As mentioned in the paper by Heidarieh (10), the immune system uses atypical chemokine receptors ACKR1, ACKR2, or ACKR4 that scavenge chemokine to control inflammation (27). These receptors are expressed mainly by erythrocytes and endothelial cells (11). ACKR1 (DARC) binds over 20 inflammatory chemokines (28) and is expressed by erythrocytes and endothelial cells (29). ACKR1 function as a scavenge receptor to limit excessive leukocyte trafficking (30). In addition, ACKR1 express on endothelial cells may reduce chemokine concentration in inflamed tissues and by that create a gradient that increases inflammation (11). ACKR2 which binds an inflammatory CC chemokines is also expressed lymphatic endothelial cells, placental trophoblasts, and leukocytes (31). ACKR2 is also a chemokine scavenger receptor (32, 33). ACKR2 control chemokine concentration by activating a β-arrestin without affecting its internalization rate (34, 35). ACKR2 promotes chemokine concentration by regulating lymphatic vessel function (36) and compactness (37), and ACKR2 KO mice in different pathological conditions show unregulated increased inflammation (38).

ACKR4 binds the homeostatic chemokines CCL19, CCL21, CCL25, and CXCL13 and is expressed by thymic epithelial cells, bronchial cells, and keratinocytes. ACKR4 is a constitutive internalization receptor with a chemokine-scavenging function (39). After chemokine binding, it recruits β-arrestin 2, but whether it activates signal transduction pathways is unknown. Few data are available on the *in vivo* role of ACKR4 in the context of inflammation. It appears to be important for the correct trafficking of dendritic cells to induce adaptive immune responses. Indeed, ACKR4 expression in lymph nodes is needed for generating a gradient of the CCR7 ligands CCL19 and CCL21 in the subcapsular sinus (40). In addition, ACKR4 KO mice were used to show that homeostatic chemokine clearance is necessary to control excessive Th17 responses that can lead to immunopathologys (41).

Herpesviruses have been shown to target the chemokine system (42, 43) by adopting and modifying chemokine and chemokine receptor genes to benefit their own survival and propagation (43–45). Interestingly, as quoted by Proudfoot et al. (46), two chemokine homologs with antagonistic activity were reported. The first one, MC148, is encoded by molluscum contagiosum virus 1, a poxvirus that infects humans and causes skin lesions lacking inflammatory cell infiltrates. MC148 does not induce by itself the migration of mononuclear cells (47, 48). However, it does inhibit the migration of immune cells mediated by CCL1, CCL2, CCL3, CCL5, CCL7, CXCL8, and CXCL12 (48, 49). The second viral chemokine, the viral macrophage inflammatory protein II (vMIP-II), also binds several chemokine receptors without inducing cell migration. vMIP-II compete with several chemokine receptors, including CCR1, CCR2, CCR5, CCR10, CXCR4, CX3CR1, and XCR1 (46).

Furthermore, in Heidarieh paper (10) it was mentioned that herpesviruses have been found to encode CKBPs to modulate the expression and function of chemokines (50–52). CKBPs were found to be expressed by non-viral pathogens. For example, a CKBP secreted by *Schistosoma mansoni* (53) interacts with chemokines such as CCL2, CCL3, CCL5, CXCL8, and CX3CL1, and inhibit their function (53). Another example is Evasins, which is expressed by ticks (54) and inhibit chemokine-induced inflammation (12). The main function of CKBPs is to inhibit chemokine activity and subsequently inhibit inflammation.

We hypothesize that lessons learned from our own immune system and from the anti-chemokine strategies that have evolved in pathogens will help us design potent and selective molecules with novel modalities that may have better success in the clinic in the future. We chose to screen for promiscuous CBPs using combinatorial libraries of random dodecapeptides (5 × 10^12^ for the linear library and 2 × 10^13^ for the circular library, of electroporated sequences) fused to a minor coat protein (pIII) of the M13 phage. We performed the screen on immobilized CCL11, CXCL9, CXCL8, CXCL12, and CCL2, and successfully identified two CBPs, BKT120 and BKT130, that could bind and inhibit the function of the chemokines CCL2 and CCL5. To improve the affinity and bioactivity of the peptides, we prepared BKT120 and BKT130 as peptide-Fc fusions protein (peptibodies). BKT130Fc peptibody bind at high affinity to inflammatory chemokines CXCL11, CXCL10, and CCL5 with K_D_ of 8.3 E−0.8, 3.8 E−0.8, and 1.8 E−0.8, respectively. BKT120Fc peptibody bind at high affinity to inflammatory chemokines CXCL10, CCL2, and CXCL9 was with K_D_ of 6.3 E−0.9, 4.6 E−0.8, and 1.8 E−0.8, respectively. Both inhibited cell adhesion in response to the inflammatory chemokines CCL2, CCL5, CXCL9, and CXCL11. BKT130Fc, also inhibited CXCL10 function, but both did not inhibit the function of the homeostatic chemokine CXCL12, the eosinophils chemokine CCL11 or the neutrophil chemokine CXCL8. Cell migration inhibition in response to the inflammatory chemokines CCL2, CCL5, CXCL10, and CXCL11 was also demonstrated and was significantly better when BKT130Fc was used. *In vivo*, BKT120Fc and BKT130Fc had a prolonged PK in mice and were inhibitory to immune-mediated, chemokine-dependent DTH. Furthermore, we also demonstrated that both could inhibit immune cell migration, inflammation, and disease progression in RA and MS mouse models. Our work has demonstrated for the first time that CBPs can be identified and developed into potential therapeutic agents in diseases that are dependent on chemokines for their progression.

## Ethics Statement

All studies were carried out in accordance with the recommendations of Hebrew University Animal Facility (Jerusalem, Israel). The protocols were approved by the Animal Care and Use Committee of Hebrew University.

## Author Contributions

MA—designed the study, performed experiments, data analysis, data interpretation, and wrote the manuscript. HW—performed experiments. DVO—performed experiments, data analysis. VG—performed experiments, data analysis. ZO—performed experiments, data analysis. AK—data analysis, data interpretation. LW—performed experiments. EG—data interpretation. AP—designed the study, data analysis, data interpretation, and wrote the manuscript. OE—designed the study, data interpretation.

## Conflict of Interest Statement

By submitting this manuscript, the corresponding author accepts the responsibility that all authors have agreed to be so listed and have observed and approved the manuscript, its content, and its submission to Leukemia. This manuscript has not been published nor is it under consideration for publication elsewhere, including Internet publication. MA and OE are employees and shareholders of Biokine Therapeutics Ltd.; AP serves as consultant for Biokine Therapeutics and is also a shareholder. All other authors have no conflicts of interest to declare.

## Supplementary Material

Figure S1 Effect of the peptibody BKT110Fc on the chemokine-induced immune cell-dependent adhesion to VCAM-1. Adhesion was measured using the laminar flow assay. The number of adherent cells resisting detachment by elevated shear forces (dyn/cm^2^) is expressed as the percentage of originally settled cells. The effects of BKT110Fc on the **(A)** CCL2-, **(B)** CCL11-, **(C)** CXCL8-, **(D)** CXCL10-induced immune cell-dependent adhesion to VCAM-1 were measured. All the adhesion experiments were performed at least three times on multiple test fields.

Figure S2 Production of antibodies against BKT130Fc. C57BL/6 mice were i.v. injected with 50 μg of BKT130Fc once or twice a week for total of four injections. One week after the last injection serum was extract and ELISA was performed. Sera were diluted at 1:5, 1:50, and 1:500 and loaded on plates that were pre-coated with **(A)** BKT130Fc, **(B)** BKT120Fc, or **(C)** BSA. The data are presented as the optical density (O.D.) obtained at wavelength of 450 nm.
