# Supplementary figures and images for: Development of Novel Promiscuous Anti-Chemokine Peptibodies for Treating Autoimmunity and Inflammation
Source: Front Immunol. 2017 Nov 23;8:1432. doi: 10.3389/fimmu.2017.01432 (PMC5703867; doi:10.3389/fimmu.2017.01432)

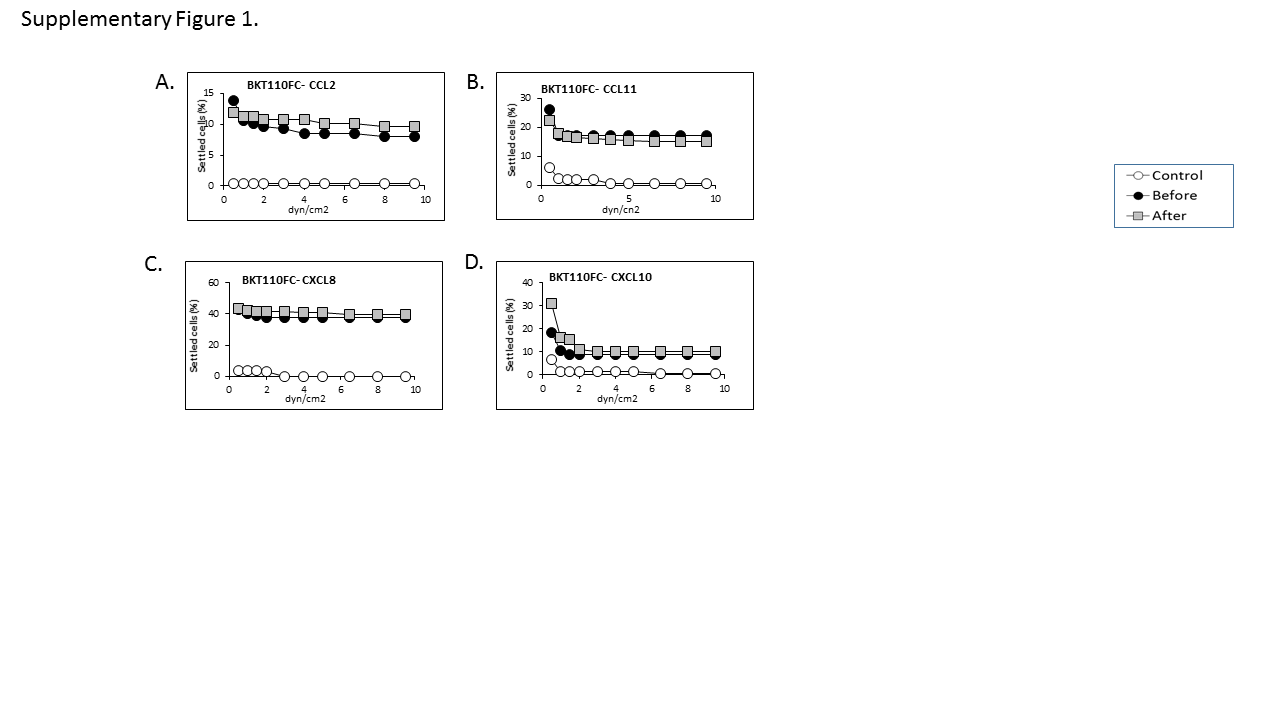

Supplement: Figure S1 — Effect of the peptibody BKT110Fc on the chemokine-induced immune cell-dependent adhesion to VCAM-1. Adhesion was measured using the laminar flow assay. The number of adherent cells resisting detachment by elevated shear forces (dyn/cm2) is expressed as the percentage of originally settled cells. The effects of BKT110Fc on the (A) CCL2-, (B) CCL11-, (C) CXCL8-, (D) CXCL10-induced immune cell-dependent adhesion to VCAM-1 were measured. All the adhesion experiments were performed at least three times on multiple test fields. [file image_1.tif]

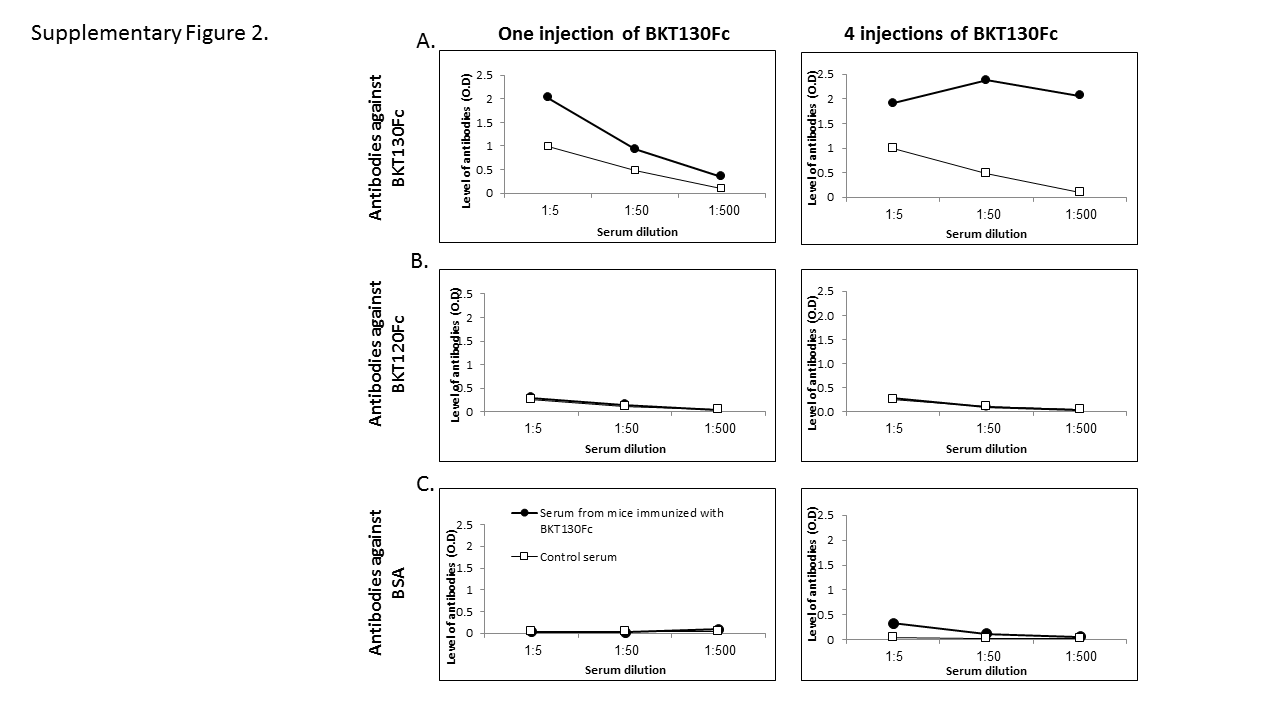

Supplement: Figure S2 — Production of antibodies against BKT130Fc. C57BL/6 mice were i.v. injected with 50 μg of BKT130Fc once or twice a week for total of four injections. One week after the last injection serum was extract and ELISA was performed. Sera were diluted at 1:5, 1:50, and 1:500 and loaded on plates that were pre-coated with (A) BKT130Fc, (B) BKT120Fc, or (C) BSA. The data are presented as the optical density (O.D.) obtained at wavelength of 450 nm. [file image_2.tif]
