# Supplementary material for: Development of Novel Promiscuous Anti-Chemokine Peptibodies for Treating Autoimmunity and Inflammation
Source: Front Immunol. 2017 Nov 23;8:1432. doi: 10.3389/fimmu.2017.01432 (PMC5703867; doi:10.3389/fimmu.2017.01432)
Supplement: Supplementary file 3 [file table_1.docx]

Supplementary Table 1: List of all the peptides, bound to CCL11, CXCL9, CXCL8, CCL2, CXCL12 fished from the two libraries.

| **Peptide Name** | **Sequence of peptide** |
| --- | --- |
| BKT-P50 | CAHLSPHKC |
| BKT-P10 | CDIPWRNEC |
| BKT-P17 | CDPLRQHSC |
| BKT-P58 | CDSLGHWLC |
| BKT-P15 | CDYTTRHSC |
| BKT-P59 | CHGTLNPEC |
| BKT-P56 | CHHNLSWEC |
| BKT-P60 | CHIWTLASC |
| BKT-P61 | CHNTFSPRC |
| BKT-P62 | CIPLHASLC |
| BKT-P63 | CITTTSLSC |
| BKT-P64 | CKLTTCKDC |
| BKT-P65 | CKNHTTFWC |
| BKT-P66 | CLKLLSRSC |
| BKT-P67 | CLLKAHPSC |
| BKT-P68 | CLNQLKQAC |
| BKT-P69 | CMNFPSPHC |
| BKT-P70 | CPQSPTYTC |
| BKT-P57 | CPSSAIHTC |
| BKT-P71 | CPTSTARIC |
| BKT-P72 | CQASSFPSC |
| BKT-P73 | CQPYFWYRC |
| BKT-P14 | CQTLTPSIC |
| BKT-P74 | CSKLGHLWC |
| BKT-P75 | CSKTPERIX |
| BKT-P76 | CSNNNRMTC |
| BKT-P77 | CSPILSLSC |
| BKT-P16 | CSPTNFTRC |
| BKT-P78 | CSRPAMNVC |
| BKT-P79 | CSTKAYPNC |
| BKT-P80 | CSTSSCGSC |
| BKT-P81 | CSYWGHRDC |
| BKT-P13 | CTAHDANAC |
| BKT-P82 | CTANSEKTC |
| BKT-P83 | CTHPKASMC |
| BKT-P84 | CTKTINGKC |
| BKT-P85 | CTNMQSPLC |
| BKT-P86 | CTPFTKLPC |
| BKT-P87 | CTPTTDSIC |
| BKT-P88 | CTQQNGHPC |
| BKT-P12 | ACTTPSKHQC |
| BKT-P89 | CTYNVAKPC |
| BKT-P90 | ACAPLMFSQC |
| BKT-P48 | ACHASLKHRC |
| BKT-P91 | AHFSPNLLLGG |
| BKT-P44 | AHSLKSITNHGL |
| BKT-P92 | AKTLMPSPFPRT |
| BKT-P93 | ASAVGSLSIRWQ/L/G |
| BKT-P94 | ASWVDSRQPSAA |
| BKT-P95 | CPQLTVGQHRT |
| BKT-P8 | DLPPTLHTTGSP |
| BKT-P96 | DSSNPIFWRPSS |
| BKT-P97 | EFLGVPASLVNP |
| BKT-P51 | ESDLTHALHWLG |
| BKT-P98 | EVHSTDRYRSIP |
| BKT-P99 | FGLQPTGDIARR |
| BKT-P9 | FSMDDPERVRSP |
| BKT-P100 | FSPLHTSTYRPS |
| BKT-P27 | GDFNSGHHTTTR |
| BKT-P28 | GPSNNLPWSNTP |
| BKT-P33 | GVHKHFYSRWLG |
| BKT-P101 | HAPLTRSPAPNL |
| BKT-P102 | HGSLTTLF/LRYEP |
| BKT-P45 | HHFHLPKLRPPV |
| BKT-P55 | HHTWDTRIWQAF |
| BKT-P54 | HPTTPFIHMPNF |
| BKT-P103 | HRDPXS(P)PSAA/GRP |
| BKT-P104 | HNVTTRTQRLMP |
| BKT-P49 | HSACHASLKHRC |
| BKT-P105 | HSACKLTTCKDG |
| BKT-P6 | HSACLSTKTNIC |
| BKT-P106 | IAHVPETRLAQM |
| BKT-P107 | IFSMGTALARPL |
| BKT-P108 | INKHPQQVSTLL |
| BKT-P7 | ISPSHSQAQADL |
| BKT-P46 | LDYPIPQTVLHH |
| BKT-21 | LFAAVPSTQFFR |
| BKT-P22/38 | LGFDPTSTRFYT |
| BKT-P37 | LLADTTHHRPWP |
| BKT-P109 | LPWAPNLPDSTA |
| BKT-P110 | LQPSQPQRFAPT |
| BKT-P111 | LSPPMQLQPTYS |
| BKT-P112 | MHNVSDSNDSAI |
| BKT-P113 | NSSMLGMLPSSF |
| BKT-P114 | NTSSSQGTQRLG |
| BKT-P42 | PGQWPSSLTLYK |
| BKT-P23 | QIPQMRILHPYG |
| BKT-P24 | QIQKPPRTPPSL |
| BKT-P115 | QLTQTMWKDTTL |
| BKT-P116 | QNLPPERYSEAT |
| BKT-P117 | QSLSFAGPPAWQ |
| BKT-P118 | QTTMTPLWPSFS |
| BKT-P119 | RCMSEVISFNCP |
| BKT-P120 | RSPYYNKWSSKF |
| BKT-P39 | SAGHIHEAHRPL |
| BKT-P40 | SAISDHRAHRSH |
| BKT-P121 | SEPTYWRPNMSG |
| BKT-P32 | SFAPDIKYPVPS |
| BKT-P31 | SFWHHHSPRSPL |
| BKT-P3 | SIFAHQTPTHKN |
| BKT-P2 | SIPSHSIHSAKA |
| BKT-P122 | SIRTSMNPPNLL |
| BKT-P123 | SLPHYIDNPFRQ |
| BKT-P29 | SLSKANILHLYG |
| BKT-P124 | SLVTADASFTPS |
| BKT-P125 | SMVYGNRLPSAL |
| BKT-P126 | SPSLMARSSPYW |
| BKT-P127 | SPNLPWSKLSAY |
| BKT-P1 | SQTLPYSNAPSP |
| BKT-P128 | SSTQAHPFAPQL |
| BKT-P129 | STPNSYSLPQAR |
| BKT-P4 | STVVMQPPPRPA |
| BKT-P34 | SVQTRPLFHSHF |
| BKT-P130 | SVSVGMKPSPRP |
| BKT-P131 | SYIDSMVPSTQT |
| BKT-P132 | SYKTTDSDTSPL |
| BKT-P133 | TAAASNLRAVPP |
| BKT-P5 | TAPLSHPPRPGA |
| BKT-P134 | TGLLPNSSGAGI |
| BKT-P135 | TGPPSRQPAPLH |
| BKT-P30 | TLSNGHRYLELL |
| BKT-P25 | TPSPKLLQVFQA |
| BKT-P136 | TPSTGLGMSPAV |
| BKT-P137 | TPVYSLKLGPWP |
| BKT-P47 | TRLVPSRYYHHP |
| BKT-P138 | TSPIPQMRTVPP |
| BKT-P139 | TTNSSMTMQLQR |
| BKT-P140 | TTTLPVQPTLRN |
| BKT-P141 | TTTWTTTARWPL |
| BKT-P142 | TVAQMPPHWQLT |
| BKT-P143 | TWNSNSTQYGNR |
| BKT-P144 | TWTLPAMHPRPA |
| BKT-P26 | VHTSLLQKHPLP |
| BKT-P35 | VLPNIYMTLSA |
| BKT-P145 | VMDFASPAHVLP |
| BKT-P146 | VNQEYWFFPRRP |
| BKT-P147 | VYSSPLSQLPR |
| BKT-P148 | VPPIS(R)TFLF(L)ST(K)S |
| BKT-P149 | VPPLHPALSRXN |
| BKT-P43 | VSPFLSPTPLLF |
| BKT-P150 | VSRLGTPSMHPS |
| BKT-P151 | WPFNHFPWWNVP |
| BKT-P52 | WSAHIVPYSHKP |
| BKT-P152 | WWPNSLNWVPRP |
| BKT-P53 | YATQHNWRLKHE |
| BKT-P153 | YCPMRLCTDC |
| BKT-P154 | YGKGFSPYFHVT |
| BKT-P155 | YPHYSLPGSSTL |
| BKT-P156 | YPSLLKMQPQFS |
| BKT-P157 | YQPRPFVTTSPM |
| BKT-P158 | YSAPLARSNVVM |
| BKT-P36 | YTRLSHNPYTLS |
| BKT-P41 | YTTHVLPFAPSS |
| BKT-P159 | YTWQTIREQYEM |
| BKT-P6 | (A)CLSTKTNI(C)  HSACLSTKTNIC |
| BKT-P11 | CTTPSKHQC |
