# Supplementary material for: Development of Novel Promiscuous Anti-Chemokine Peptibodies for Treating Autoimmunity and Inflammation
Source: Front Immunol. 2017 Nov 23;8:1432. doi: 10.3389/fimmu.2017.01432 (PMC5703867; doi:10.3389/fimmu.2017.01432)
Supplement: Supplementary file 4 [file table_2.docx]

Supplementary Table 2: List of CCL2-binding peptides, fished from the two libraries.

| **Peptide Name** | **Peptide Sequence** | **No. of clones identified** |
| --- | --- | --- |
| BKT120 (Pep 46) | LDYPIPQTVLHH | 1 |
| BKT130 (Pep 2) | SIPSHSIHSAKA | 12 |
| PEP 37 | LLADTTHHRPWP | 1 |
| PEP 24 | QIQKPPRTPPSL | 2 |
| PEP 28 | GPSNNLPWSNTP | 1 |
| PEP 6 | (A)CLSTKTNI(C) | 6 |
| PEP 48 | ACHASLKHRC | 1 |
| PEP 92 | AKTLMPSPFPRT | 1 |
| PEP 95 | CPQLTVGQHRT | 1 |
| PEP 9 | FSMDDPERVRSP | 1 |
| PEP 51 | ESDLTHALHWLG | 1 |
| PEP 97 | EFLGVPASLVNP | 1 |
| PEP 27 | GDFNSGHHTTTR | 1 |
| PEP 101 | HAPLTRSPAPNL | 1 |
| PEP 102 | HGSLTTLF/LRYEP | 1 |
| PEP 55 | HHTWDTRIWQAF | 1 |
| PEP 103 | HRDP()SP(P)SAA/GRP | 1 |
| PEP 104 | HNVTTRTQRLMP | 1 |
| PEP 107 | IFSMGTALARPL | 1 |
| PEP 110 | LQPSQPQRFAPT | 1 |
| PEP 111 | LSPPMQLQPTYS | 1 |
| PEP 114 | NTSSSQGTQRLG | 1 |
| PEP 40 | SAISDHRAHRSH | 1 |
| PEP 45 | HHFHLPKLRPPV | 1 |
| PEP 47 | TRLVPSRYYHHP | 1 |
| PEP 121 | SEPTYWRPNMSG | 1 |
| PEP 32 | SFAPDIKYPVPS | 1 |
| PEP 3 | SIFAHQTPTHKN | 1 |
| PEP 122 | SIRTSMNPPNLL | 1 |
| PEP 123 | SLPHYIDNPFRQ | 1 |
| PEP 126 | SPSLMARSSPYW | 1 |
| PEP 128 | SSTQAHPFAPQL | 1 |
| PEP 4 | STVVMQPPPRPA | 1 |
| PEP 130 | SVSVGMKPSPRP | 1 |
| PEP 131 | SYIDSMVPSTQT | 1 |
| PEP 133 | TAAASNLRAVPP | 1 |
| PEP 5 | TAPLSHPPRPGA | 1 |
| PEP 135 | TGPPSRQPAPLH | 1 |
| PEP 30 | TLSNGHRYLELL | 1 |
| PEP 136 | TPSTGLGMSPAV | 1 |
| PEP 139 | TTNSSMTMQLQR | 1 |
| PEP 140 | TTTLPVQPTLRN | 1 |
| PEP 141 | TTTWTTTARWPL | 1 |
| PEP 26 | VHTSLLQKHPLP | 1 |
| PEP 145 | VMDFASPAHVLP | 1 |
| PEP 148 | VPPIS(R)TFLF(L)ST(K)S | 1 |
| PEP 43 | VSPFLSPTPLLF | 1 |
| PEP 52 | WSAHIVPYSHKP | 1 |
| PEP 53 | YATQHNWRLKHE | 1 |
| PEP 154 | YGKGFSPYFHVT | 1 |
| PEP 159 | YTWQTIREQYEM | 1 |
| PEP 16 | CSPTNFTRC | 1 |
| PEP 21 | LFAAVPSTQFFR | 1 |
| PEP 44 | AHSLKSITNHGL | 1 |
| PEP 49 | HSACHASLKHRC | 1 |
| PEP 58 | CDSLGHWLC | 1 |
| PEP 64 | CKLTTCKDC | 1 |
| PEP 74 | CSKLGHLWC | 1 |
| PEP 76 | CSNNNRMTC | 1 |
| PEP 79 | CSTKAYPNC | 1 |
| PEP 80 | CSTSSCGSC | 1 |
| PEP 82 | CTANSEKTC | 1 |
| PEP 84 | CTKTINGKC | 1 |
| PEP 85 | CTNMQSPLC | 1 |
| PEP 105 | HSACKLTTCKDG/C | 1 |
| PEP 120 | RSPYYNKWSSKF | 1 |
| PEP 71 | CPTSTARIC | 1 |
| PEP 75 | CSKTPERIC | 1 |
| PEP 153 | YCPMRLCTDC | 2 |
| neglin | QMGTTLPGILRN | 1 |
