# Supplementary material for: Development of Novel Promiscuous Anti-Chemokine Peptibodies for Treating Autoimmunity and Inflammation
Source: Front Immunol. 2017 Nov 23;8:1432. doi: 10.3389/fimmu.2017.01432 (PMC5703867; doi:10.3389/fimmu.2017.01432)
Supplement: Supplementary file 5 [file data_sheet_1.docx]

Supplementary Figure 1: Effect of the peptibody BKT110Fc on the chemokine-induced immune cell-dependent adhesion to VCAM-1. Adhesion was measured using the laminar flow assay. The number of adherent cells resisting detachment by elevated shear forces (dyn/cm^2^) is expressed as the percentage of originally settled cells. The effects of BKT110Fc on the (A) CCL2-, (B) CCL11, (C) CXCL8-, (D) CXCL10 -induced immune cell-dependent adhesion to VCAM-1 were measured. All the adhesion experiments were performed at least three times on multiple test fields.

Supplementary Figure 2: Production of antibodies against BKT130Fc. C57BL/6 mice were iv injected with 50 ug of BKT130Fc once or twice a week for total of 4 injections. One-week after the last injection serum was extract and ELISA was performed. Sera were diluted at 1:5, 1:50 and 1:500 and loaded on plates that were pre-coated with (A) BKT130Fc, (B) BKT120Fc or (C) BSA. The data are presented as the optical density (O.D.) obtained at wavelength of 450 nm.
